# Elucidating the Role of Trem2 in Lipid Metabolism and Neuroinflammation

**DOI:** 10.1111/cns.70338

**Published:** 2025-04-09

**Authors:** Chenhui Zhao, Wei Qi, Xiaoping Lv, Xueli Gao, Chaonan Liu, Shimin Zheng

**Affiliations:** ^1^ College of Veterinary Medicine Northeast Agricultural University Harbin China; ^2^ Heilongjiang Key Laboratory of Laboratory Animals and Comparative Medicine Harbin China; ^3^ Suzhou Frontage New Drug Development Co., Ltd. Suzhou China

**Keywords:** Alzheimer's disease, astrocytes, lipid metabolism, neuroinflammation, Trem2

## Abstract

**Background:**

Alzheimer's disease (AD) is a neurodegenerative disorder characterized by cognitive impairment and neuroinflammation. Astrocytes play a key role in the neuroinflammatory environment of AD, especially through lipid metabolism regulation. However, the mechanisms by which astrocytes, particularly through the triggering receptor expressed on myeloid cells 2 (Trem2) receptor, contribute to lipid dysregulation and neuroinflammation in AD remain inadequately understood.

**Methods:**

We employed an AD mouse model and integrated single‐cell RNA sequencing (scRNA‐seq), transcriptomics, and high‐throughput metabolomics to analyze lipid metabolism and inflammatory profiles in astrocytes. Differential gene expression was further validated with the GEO database, and in vitro and in vivo experiments were conducted to assess the impact of Trem2 modulation on astrocytic inflammation and lipid composition.

**Results:**

Our findings demonstrate that Trem2 modulates lipid metabolism in astrocytes, affecting fatty acid and phospholipid pathways. In the AD model, Trem2 expression was suppressed, enhancing nuclear factor‐κB (NF‐κB) signaling and promoting the secretion of pro‐inflammatory factors such as tumor necrosis factor‐α (TNF‐α) and interleukin‐6 (IL‐6). Trem2 overexpression reduced astrocytic inflammation and altered lipid composition, attenuating neuroinflammation both in vitro and in vivo. These results underscore Trem2's regulatory role in lipid metabolism and its significant impact on neuroinflammation in AD.

**Conclusions:**

This study identifies Trem2 as a pivotal regulator of astrocytic lipid metabolism and neuroinflammation in AD, providing potential molecular targets for early intervention and therapeutic strategies aimed at mitigating AD progression.

## Graphical abstract

Trem2 regulates astrocytic lipid metabolism and reduces neuroinflammation in Alzheimer's disease, highlighting a potential therapeutic target for early intervention and mitigation of AD progression.

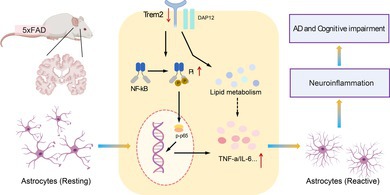

## 1 Introduction

Alzheimer's disease (AD) is a debilitating neurodegenerative disorder that has an impact on millions of individuals globally, with a particular prevalence among the elderly [1, 2, 3]. The primary symptoms of this illness encompass memory loss, cognitive impairment, as well as emotional fluctuations [4, 5, 6]. Due to the intensification of global population aging, the incidence and prevalence of AD are steadily rising. This trend pressures public health and economic development [7]. The cause of AD has not been fully understood. Numerous studies suggest that it is likely to result from a combination of intricate genetic and environmental factors, including gene variants such as APOE ε4 and chronic conditions like hyperlipidemia and diabetes [8, 9]. Neuroinflammation and abnormal lipid metabolism are pathological characteristics of AD. Gaining insights into the mechanism of its onset could enhance efforts toward the prevention and treatment of the disease [10].

Astrocytes play a crucial role in the central nervous system as principal support cells, aiding in the maintenance of neuron homeostasis, regulation of synaptic transmission, and participation in neuroinflammation regulation [11, 12, 13]. Recent studies have found that the impaired functioning of astrocytes may play a crucial role in the pathogenesis of neurodegenerative conditions like AD [14, 15, 16]. Among these, the dysregulation of lipid metabolism is considered a manifestation of astrocyte dysfunction [17, 18]. Nevertheless, the mechanisms underlying the impact of this atypical lipid metabolism on the progression of neuroinflammation and neurodegenerative diseases remain incompletely elucidated [19].

The triggering receptor expressed on myeloid cells 2 (Trem2) is primarily expressed on immune cells and plays a crucial role in maintaining tissue homeostasis by regulating cellular phagocytosis and the inflammatory response [20, 21]. Recent studies have revealed that mutations or alterations in Trem2 are associated with the onset and progression of various neurodegenerative diseases, including AD [22, 23, 24]. Additionally, TREM2 is expressed in other cell types within the central nervous system, such as astrocytes [25]. Therefore, Trem2 becomes a crucial target for investigating the pathological mechanisms of neurodegenerative diseases and exploring novel therapeutic strategies [20]. While some studies have elucidated the role of Trem2 in neuroinflammation regulation and neuronal function, a majority have focused on its role and functionality in microglial cells [21, 26, 27]. The question of how Trem2 affects astrocytic lipid metabolism, thereby impacting neuroinflammation and the pathogenesis of AD, remains unanswered [25].

Therefore, the objective of this study is to clarify the regulatory role of Trem2‐mediated astrocytic lipid metabolism in the neuroinflammation of the microenvironment associated with AD. In this study, we employed single‐cell sequencing and metabolomics techniques to examine the molecular‐level phenotypic alterations of astrocytes in AD and assess their effects on neuroinflammation. Our research not only provides novel insights into the pathological mechanisms of AD but also lays crucial theoretical groundwork for the development of innovative therapeutic strategies, specifically focusing on neuroinflammation and abnormalities in lipid metabolism.

## 2 Materials and Methods

### 2.1 Ethical Statement

All animal experiments were conducted in compliance with internationally recognized animal welfare standards and ethical guidelines. All animals were housed in environments adhering to good laboratory practice (GLP) guidelines, with sufficient food and water provided. All surgical procedures and treatments were conducted under veterinary supervision to minimize pain and discomfort. After the experiment, ethical treatment of the mice was ensured. The protocol for animal experimentation was approved by the Institutional Animal Care and Use Committee (IACUC).

### 2.2 AD Model Mice

Fifteen 5xFAD AD model mice with a C57BL/6J background, six C57BL/6J mice, and three newborn C57/BL6J mice aged 1–2 days old were purchased from Changzhou Cavens Experimental Animals Co. Ltd. Male mice between 6 and 7 months old, weighing 30 to 35 g, were utilized in the experiment. The control group consisted of C57BL/6J mice (NC group), while the experimental group comprised 5xFAD mice (the AD group). All mice were kept in SPF‐grade animal facilities at a temperature of 23°C ± 1°C, humidity of 55% ± 5%, and a 12‐h light–dark cycle [28].

### 2.3 Morris Water Maze Experiment

The Morris water maze was used to evaluate the learning and memory abilities of mice. The maze consisted of a circular pool (120 cm diameter, 30 cm depth) divided into four quadrants. A circular platform (9 cm diameter) wrapped in white gauze was placed at the center of one quadrant, submerged 1 cm below the water surface. White pigment powder was added to the water and mixed thoroughly to prevent debris from accumulating on the surface. The water temperature was maintained at 23°C ± 1°C. Visual reference objects, both proximal and distal, were fixed around the pool. A camera installed above the pool collected data, which was analyzed using video analysis software.

The experiment followed procedures from previous studies [29]. Mice were acclimated to the maze by being placed at the entrance for 30 min. The experiment consisted of two phases: acquisition and probe. During the acquisition phase, mice used visual cues to locate and memorize the hidden platform's position. Each mouse was placed in the pool facing the inner wall and allowed to swim for 90 s. If the mouse reached the platform and stayed on it for more than 2 s, the trial ended, and the latency was recorded. If the mouse failed to reach the platform or did not stay on it for 2 s, it was guided to the platform and required to remain on it for 15 s.

The acquisition phase involved 6 days of training, with experiments conducted daily at a fixed time. Each mouse underwent four training sessions per day, starting from different quadrants in sequence, with at least 15 s of rest between trials. Twenty‐four hours after the acquisition phase, the probe phase was conducted. The platform was removed, and the mouse was released from the opposite quadrant. Mice swam freely for 90 s, and the number of crossings over the original platform position and the time spent in the target quadrant were recorded. Finally, a cue test was performed by placing a platform with a red flag in the opposite quadrant. The mouse was released from the side opposite the red flag, and the time taken to find the platform was recorded.

### 2.4 Sulfur Staining

Brain tissue was collected by anesthetizing mice with an intraperitoneal injection of 0.75% sodium pentobarbital (P3761, Sigma‐Aldrich, USA). The abdominal skin was incised to expose the heart. Saline was slowly infused into the left ventricle until the right atrial appendage filled with fluid, at which point the right atrial appendage was severed. The infusion rate was then increased to flush the liver and kidneys until they turned white. Saline was replaced with 4% paraformaldehyde (P6148, Sigma‐Aldrich, USA). Perfusion was complete when the tail curled, limbs stiffened, and the body became rigid. The meninges were peeled off, and the brain was removed, placed in an EP tube with 4% paraformaldehyde, and fixed overnight at 4°C.

The brains were washed with distilled water for 2 h, dehydrated in 30% sucrose solution (718033, Sigma‐Aldrich, USA) at 4°C until fully submerged, and this process was repeated. Tissue sections were prepared using OCT embedding medium (4583, SAKURA, USA) to form circular flat bases. The brain was removed from the sucrose solution, dried with filter paper, and dissected to remove the cerebellum. The brain was fixed on the OCT base and frozen in a cryostat for 20–30 min. Sections 30 μm thick were cut at −22°C, focusing on the hippocampal region. Every sixth section was collected, placed into EP tubes, and stored at −80°C.

Thioflavin S (1326‐12‐1, Sigma‐Aldrich, USA) was used to stain brain sections for detecting senile plaques. A 1% Thioflavin S solution was prepared in 70% ethanol. Brain sections were incubated in the solution at room temperature for 5 min, then washed three times each with 50% ethanol, 70% ethanol, and 1 × PBS for 5 min each. Sections were air‐dried, mounted with DAPI‐containing medium, and covered with a coverslip. Staining results were observed using confocal microscopy [30].

### 2.5 Neurofilament Light (NfL) Detection for Serum Neurofilament Light Chain

Mice were anesthetized with an intraperitoneal injection of 0.75% pentobarbital sodium. Blood was collected from the orbital sinus and carefully allowed to flow along the walls of an EP tube to prevent cell lysis. After a 10‐min incubation, the blood samples were centrifuged at 10,000 *g* for 5 min at 4°C. The supernatant was gently removed and transferred to a pre‐cooled EP tube. The serum was diluted fourfold, and NfL concentration was measured using single‐molecule array technology. Following the reagent manufacturer's protocol, the Simoa HD‐1 Analysis Platform (Simoa HD‐1, Quanterix, USA) and a single NfL reagent kit (104073, Quanterix, USA) were used. Magnetic beads pre‐coupled with antibodies were utilized to capture NfL, forming enzyme‐labeled immunocomplexes. Non‐specifically bound proteins were removed using a wash solution, and an enzyme substrate was added. The beads and substrate were transferred to a SimoaTM disc, where they settled into microcavities and were sealed with oil. Substrate catalysis by β‐galactosidase generated resorufin, and the signal was detected using a CCD camera. The luminescence intensity was analyzed to determine serum NfL concentration [31].

### 2.6 Single‐Cell RNA‐Seq

Brain tissues were collected from three euthanized mice in both the AD and NC groups. The tissues were enzymatically dissociated using pancreatic enzymes (T2600000, Sigma, USA) and DNase I (11284932001, Sigma, USA), as provided by BD. Single cells were isolated using Ficoll‐Paque PLUS (GE Healthcare) through density gradient centrifugation. The single‐cell suspensions were processed with the Chromium Single Cell 3′ Solution from 10× Genomics to construct individual cell cDNA libraries. Transcriptomic data for each cell were obtained using high‐throughput sequencing on the Illumina NovaSeq 6000 platform. The resulting data were analyzed using bioinformatics methods [32].

### 2.7 Analysis of Single‐Cell RNA Sequencing (scRNA‐Seq) Data

The analysis of scRNA‐seq data was conducted using the “Seurat” package in R (version: v4.1.1). First, a series of quality control steps were applied to the data. The quality control filtering thresholds were set as follows: nFeature_RNA > 500, nCount_RNA > 1000, nCount_RNA < 20,000, and percent.mt < 10. Subsequently, the canonical correlation analysis (CCA) method was used to eliminate batch effects, and the data was normalized using the LogNormalize function.

Next, principal component analysis (PCA) was used to select key principal components, and further analysis was performed using t‐SNE clustering. To identify marker genes for cell clusters, the SingleR package was used in combination with a reference dataset, leveraging the CellMarker database (http://xteam.xbio.top/CellMarker/index.jsp) for cell annotation. Differentially expressed genes between the cells of interest and other cells were designated as marker genes for those clusters.

Additionally, pseudo‐temporal analysis was performed using the “monocle” package, and the DDRTree algorithm was employed for dimensionality reduction. Cells were ordered based on the expression trends of sorting genes, and trajectory construction was ultimately completed [33].

### 2.8 Transcriptome Sequencing of the Mouse Cerebral Cortex and Hippocampus Tissues

Brain cortex and hippocampal tissues were collected from three euthanized mice in each group after intraperitoneal injection of 3% pentobarbital sodium. Frozen tissues were pulverized in liquid nitrogen using a mortar and pestle. The total RNA was extracted, and the MGIEasy rRNA Removal Kit (1000005953, MGI, China) was used to remove rRNA. RNA was fragmented into approximately 300 base pairs (bp) through ion shearing. First‐strand cDNA was synthesized using a random primer and reverse transcriptase, followed by the synthesis of second‐strand cDNA. Library fragments of approximately 450 bp were enriched through PCR amplification.

Library quality was assessed using the Agilent 2100 Bioanalyzer to determine total and effective concentrations. Libraries with unique indexes were pooled based on their effective concentrations and desired data output. The pooled library was diluted to 2 nM and converted into a single‐stranded library through alkaline denaturation.

Next‐generation sequencing (NGS) was performed on the Illumina HiSeq platform. Sequencing adapter sequences were removed using cutadapt (v1.18), and low‐quality sequences were filtered using fqtools (v0.1.6), yielding high‐quality clean reads. The Q30 quality value was calculated for the dataset. Clean reads were aligned to the mouse genome ( *Mus musculus* GRCm38.90) using HiSAT2 (v.2.1.0).

Differential analysis of RNA was performed using the “DESeq” R package. Genes with an absolute log fold change > 1 and a *p*‐value < 0.05 were identified as differentially expressed genes (DEGs) associated with AD. Heat maps and volcano plots of DEGs were generated using the “Pheatmap” R package. Further analysis of cortical and hippocampal DEGs was conducted using the Xiantao Academic Online Analysis Database to identify key genes implicated in AD progression [34].

### 2.9 Gene Ontology (GO)/Kyoto Encyclopedia of Genes and Genomes (KEGG) Functional Enrichment Analysis

Marker genes for key cell types were identified from differential expression and intersection analyses of scRNA‐seq data. Functional enrichment analysis was conducted using the “ClusterProfiler” package in R software, incorporating the GO and KEGG databases for mouse‐specific analyses. Enriched biological processes (BP), cellular components (CC), molecular functions (MF), and metabolic pathways in DEGs were identified. Results from GO and KEGG analyses were filtered using a significance threshold of *p* < 0.05. Further analysis evaluated the roles of key genes in cellular functions and signaling pathways, with enriched genes extracted from the GeneCards database. Marker genes, DEGs, and diagnostic ROC curves were integrated to identify critical factors contributing to AD pathogenesis, and their regulatory mechanisms were predicted [35].

### 2.10 Differential Analysis in the GEO Database

The AD‐related microarray dataset GSE165111 was retrieved from the GEO database (https://www.ncbi.nlm.nih.gov/geo/). Gene expression data from the hippocampus (HIPP) and whole brain tissues of the AD model and wild‐type (WT) mice were extracted and annotated using Perl scripts. Differential expression analysis was performed separately for HIPP and brain data using the “Limma” package in R, with genes showing *p* < 0.05 considered significant. Trem2 expression levels were extracted and examined for differential expression between the AD and WT groups [36].

### 2.11 High‐Throughput Metabolomics Sequencing and Data Analysis

Mice were anesthetized with intraperitoneal injections of 0.75% pentobarbital sodium. Cerebrospinal fluid (CSF) was collected by puncturing the subarachnoid space with a needle and gently aspirating it into a syringe. CSF samples were stored at −80°C. After thawing, 100 μL of each CSF sample was mixed with 900 μL of 80% methanol containing 0.1% formic acid, vortexed for 2 min, and centrifuged at 12,000 *g* for 10 min. The supernatant was transferred to an auto‐sampler vial.

Plasma metabolomics was conducted using an LC20 ultra‐high‐performance liquid chromatography (UHPLC) system (Shimadzu, Japan) coupled with a Triple TOF‐6600 mass spectrometer (AB Sciex). Chromatographic analysis utilized a Waters ACQUITY UPLC HSS T3 C18 column (100 × 2.1 mm, 1.8 μm) at 40°C with a flow rate of 0.4 mL/min. The mobile phase consisted of acetonitrile with 0.1% formic acid, with gradient elution as follows: 5% from 0.0 to 11.0 min, 90% from 11.1 to 12.0 min, and 5% from 12.1 to 14.0 min. The eluent was directly introduced into the mass spectrometer without fragmentation [37].

Mass spectrometry conditions included an ionization voltage of 5500 V, a capillary temperature of 550°C, a spray gas flow rate of 50 psi, and an auxiliary gas flow rate of 60 psi. Orthogonal Partial Least Squares Discriminant Analysis (OPLS‐DA) and 100 permutation tests were performed to minimize overfitting. Metabolites with VIP scores > 1 were classified as differential metabolites. Univariate analysis identified metabolites with fold changes ≥ 2 and *p* < 0.05 (Student's *t*‐test). Metabolic pathways associated with these metabolites were identified using MetaboAnalyst (version 5.0) [38].

### 2.12 Isolation and Culture of Star‐Shaped Glial Cells

Neonatal C57BL/6J mice (1–2 days old) were briefly submerged in 75% ethanol for sterilization and then transferred to a sterile workbench. After anesthesia with intraperitoneal injections of 3% pentobarbital sodium, the heads were removed, and the brains were dissected in PBS at 4°C. The cerebellum and blood vessels were removed, leaving only the cerebral cortex, which was rinsed three times with PBS.

The cortical tissue was digested with trypsin containing EDTA (T4049, Sigma, USA) at 37°C for 10–15 min, with gentle agitation every 3–5 min. An equal volume of complete culture medium was added to stop digestion. The tissue was pipetted to create a suspension and centrifuged at 112 × g for 10 min at 4°C. After removing the supernatant, the pellet was resuspended in complete medium and filtered through a 70 μm mesh to remove debris. The filtrate was centrifuged again, and the pellet was resuspended in fresh complete medium (SLM‐246, Sigma, USA).

The cell suspension was seeded in PDL‐coated culture flasks to remove fibroblasts using differential adhesion. After 1 h, non‐adherent cells were removed, and the medium was replaced. The medium was refreshed daily, and cell density was monitored. When the culture reached confluence (7–10 days), cell purification was performed by shaking the flask at 37°C and 260 rpm for 4–6 h to remove small adherent cells. Digestion and subculturing further purified the astrocytes.

Astrocyte morphology was observed using an inverted microscope, and GFAP expression was detected by immunofluorescence staining [39].

### 2.13 Western Blot

Proteins were extracted from cells or tissues using RIPA buffer (P0013B; Shanghai Beitu Bio, China), and the protein concentration was determined with the BCA Protein Quantification Kit (A53226; ThermoFisher, USA). Next, the protein was separated and transferred onto a polyvinylidene fluoride (PVDF) membrane using the sodium dodecyl sulfate‐polyacrylamide gel electrophoresis (SDS‐PAGE) method with IPVH85R equipment from Millipore in Germany. The membrane was blocked by incubating it with 5% BSA at room temperature for 1 h and subsequently exposed to immunoblotting using the appropriate primary and secondary antibodies. The membrane was washed with TBST for 5 min, repeated three times, and then tested using a chemiluminescent detector. Protein quantification analysis was conducted using ImageJ 1.48u software (V1.48, National Institutes of Health, USA). This analysis relied on the grayscale ratio of each protein compared to the internal reference protein β‐actin. The sources of antibodies used in this study are specified in Table S1 [40, 41].

### 2.14 ELISA

Inflammatory factors TNF‐α, IL‐6, and interleukin‐1β (IL‐1β) were detected using ELISA kits according to the manufacturer's instructions. Briefly, 50 μL of each sample was added to a 96‐well plate and incubated at 37°C in a CO_2_‐humidified incubator for 24 h. Absorbance at 450 nm was measured using a multimode microplate reader (Synergy 2, BioTek, USA). The standard curve regression equation was constructed by plotting standard concentrations (*x*‐axis) against absorbance values (*y*‐axis). Sample absorbance values were substituted into the equation to calculate the target protein concentration. The origin of the ELISA kit used in this study is listed in Table S1 [42].

### 2.15 Immunofluorescence Staining

Samples were washed three times in 1 × PBS for 5 min each. A blocking solution containing 5% BSA and 0.1% Triton X‐100 was applied and incubated on a shaker at room temperature for 1.5 h. After washing three times with 1 × PBS, the samples were incubated with primary antibody overnight (14–16 h) at 4°C. Following three additional washes with 1 × PBS, the samples were incubated with secondary antibody at room temperature in the dark for 1.5 h. The samples were then mounted on glass slides, sealed with DAPI‐containing medium, and covered with coverslips. Care was taken to avoid air bubbles between tissue sections and coverslips. Fluorescent microscopy (Leica) was used for imaging. Antibody sources are detailed in Table S1 [40, 41].

### 2.16 RT‐qPCR

Total RNA was extracted from cells using TRIzol (15596026, ThermoFisher, USA). The concentration and purity of the extracted RNA were measured by utilizing a NanoDrop 2000 spectrophotometer (ND‐2000, ThermoFisher, USA). The reverse transcription synthesis of mRNA into cDNA followed the instructions from the PrimeScript RT kit (RR047A, Takara, Japan). The synthesized cDNA was employed for RT‐qPCR detection utilizing the Fast SYBR Green PCR kit (11736059, ThermoFisher, USA), with three replicates assigned to each well. When β‐actin is used as the reference gene, 2^−ΔΔCt^ indicates the fold change in gene expression between the experimental and control groups. The formula for ΔΔCT is as follows: ΔΔCT = ΔCt experimental group—ΔCt control group, where ΔCt = Ct target gene—Ct reference gene. Ct represents the amplification cycle number when the real‐time fluorescence intensity reaches the set threshold, indicating exponential growth. Experiments were repeated three times. The primer sequences are provided in Table S2 [43].

### 2.17 Cell Transfection and Treatment

Lentiviral vectors overexpressing Trem2 or DAP12, marked with GFP, were designed and synthesized by Suzhou Genbase Biotechnology Co. Ltd. (oe‐Trem2/oe‐DAP12 group; viral titer: 10^8^ TU). Negative control vectors (NC group) were used as controls. Primary astrocytes were divided into three groups: NC, oe‐Trem2, and oe‐DAP12. Cells were infected at 50% confluency and screened with 10 μg/mL puromycin (540222, Sigma‐Aldrich, USA) for 48 h. Screening continued for at least 1 week to identify stable transfected cells.

To mimic Trem2 activation, astrocytes were incubated with 5 μg/mL rabbit anti‐Trem2 antibody (ab305103, Abcam, UK), or irrelevant rabbit IgG (ab172730, Abcam, UK) for 30 min, followed by incubation with 2.5 μg/mL secondary antibody (ab150077, Abcam, UK) for 1 h. Cells were treated with 25 ng/mL lipopolysaccharide (LPS, L5293, Sigma‐Aldrich, USA) for 4 h to induce inflammation. After stimulation, RNA and proteins were extracted, and gene expression was analyzed using RT‐qPCR, western blot, and immunofluorescence [44].

### 2.18 Untargeted Lipidomics

Oe‐Trem2 cell pools (~10 million cells) were stimulated with 25 ng/mL LPS for 4 h. Lipids were extracted using the methyl tert‐butyl ether (MTBE) method. Briefly, 200 μL of water was added to the sample and vortexed for 5 s. Next, 240 μL of pre‐chilled methanol was added and vortexed for 30 s. Subsequently, 800 μL of MTBE (34875, Sigma‐Aldrich, USA) was added, followed by sonication for 20 min at 4°C. The mixture was left at room temperature for 30 min before centrifugation at 14,000 *g* for 15 min to separate the upper organic solvent layer. The organic phase was dried under a nitrogen stream. The lipid extract was dissolved in 200 μL of 90% acetonitrile and centrifuged at 14,000 *g* for 15 min. 3 μL of sample was injected for analysis. Differential lipid metabolites were identified using liquid chromatography‐mass spectrometry (LC–MS) combined with multivariate statistical analysis as previously described [45].

### 2.19 Stereotaxic Injection Into the Mouse Brain to Modulate the Expression of Trem2

Male AD mice aged 7–8 months were selected and randomly divided into three groups (10 mice per group, *N* = 10): the AD group (5xFAD AD model mice), the AD + NC group (5xFAD AD model mice injected with 0.5 μL [10^12^ TU] of control adeno‐associated virus [AAV] into the brain), and the AD + AAV group (5xFAD AD model mice injected with 0.5 μL [10^12^ TU] of Trem2‐interfering AAV into the brain). To knock down the Trem2 gene, Trem2‐interfering AAV and control AAV were designed and synthesized by Suzhou GenePharma Co. Ltd. (Suzhou, China).

Mice were anesthetized by intraperitoneal injection of 0.75% sodium pentobarbital, and the hair on their heads was shaved. The mouse's head was then secured in a stereotaxic apparatus. The skin of the head was incised to expose the skull. Using the bregma as a reference point, the plane was adjusted, and the injection sites were located in the bilateral dentate gyrus (DG) region (AP: −2.0, ML: ±1.2, DV: 2.0). The AAV was slowly injected at a volume of 0.5 μL (10^12^ TU). The injection needle was left in place for 5 min before being carefully withdrawn, and the scalp was sutured.

Twenty‐eight days post‐injection, mice were euthanized, and DG tissues were collected for analysis using protein blotting and qPCR to evaluate Trem2 interference. Behavioral tests, including the open‐field test and Y‐maze, were conducted. Tissues were further analyzed for amyloid burden and inflammatory factor expression using techniques such as immunofluorescence and protein blotting [46].

### 2.20 Behavioral Test

The open‐field test was used to evaluate the spontaneous behavior and mental state of mice in a novel environment. The open field box measured 30 × 40 × 40 cm, with a camera mounted above to track mouse movements. Mice were gently handled twice daily for 1 min each session before the experiment. Prior to testing, they were placed in the experimental room for 3 min to acclimate. Mice were then placed at the center of the box and allowed to explore freely for 5 min. After each session, the box was cleaned with alcohol and air‐dried to avoid odor contamination from previous subjects. Movement trajectory data were used to calculate the distance traveled and the time spent in the center of the box.

The Y‐maze test assessed exploratory behavior and spatial memory. The maze consisted of three arms (35 × 5 × 10 cm) arranged at 120° angles. A camera above the maze recorded mouse movements. A screen was placed between the maze and the experimenter to minimize external interference. Mice were acclimated to the experimental room for 30 min before testing. Arms were labeled A, B, and C. Mice were placed in arm A, facing the maze, and allowed to explore freely for 5 min. The number of entries into each arm was recorded.

The Morris water maze was conducted as described earlier [47, 48].

### 2.21 Statistical Analysis

During the research process, we utilized suitable statistical analysis methods to manage all the collected data. Descriptive statistical methods, such as the mean, standard deviation, and standard error, are employed to provide a summary and description of the data. The Shapiro–Wilk test determines if the data follow a normal distribution. To assess the significance of intergroup differences in data that conform to a normal distribution, we employ the Student's *t*‐test to compare two groups and analysis of variance (ANOVA) to compare multiple groups. If the data does not conform to a normal distribution, we will employ relevant non‐parametric testing techniques, such as the Mann–Whitney *U* test or the Kruskal–Wallis test. To compare count data or categorical data, we will employ either the Chi‐square test or Fisher's exact test. In multivariable analysis, logistic or linear regression models adjust for confounding factors as covariates. Statistical analyses were conducted using the R software (R Foundation for Statistical Computing) or SPSS (IBM Corp). All tests were two‐sided, and a *p*‐value of less than 0.05 was considered statistically significant.

## 3 Results

### 3.1 Establishing the Validity of the 5xFAD Mouse Model for AD Research Through Behavioral Assessment, Biomarker Analysis, and Single‐Cell Sequencing

Neuroinflammation is one of the major contributors to the progression of AD [49]. To investigate the mechanisms underlying neuroinflammation, we utilized the 5xFAD AD mouse model based on previous studies [50, 51, 52, 53] to study the development and progression of AD. Currently, approximately 10% of AD research studies employ this model (AlzPED). To validate the similarity between the 5xFAD mouse model and AD pathology, we conducted Morris water maze behavioral tests to evaluate the learning and memory abilities of mice in the model group (AD group) and control group (NC group). Additionally, AD‐specific markers, such as senile plaques and serum neurofilament light chain (NfL) levels, were detected using Thioflavin S staining and single‐molecule array technology, respectively [53]. The water maze experiments showed a gradual decrease in the latency to find the platform in all groups of mice over a 5‐day training period. As time progressed, the AD group displayed a prolonged escape latency compared to the NC group (Figure S1A). During the stage of space exploration, the mice in the AD group exhibited a lower number of platform crossings compared to the NC group (Figure S1B). The results of staining with sulfur yellow S and detection using single‐molecule array technology showed an increase in the number of senile plaques in the hippocampal dentate gyrus (Figure S1C) and serum Nfl levels (Figure S1D) in the AD group of mice compared to the NC group of mice. The above findings suggest that the 5xFAD mouse model exhibits characteristics that are generally consistent with those of AD, making it suitable for further experimental use.

**FIGURE 1:**
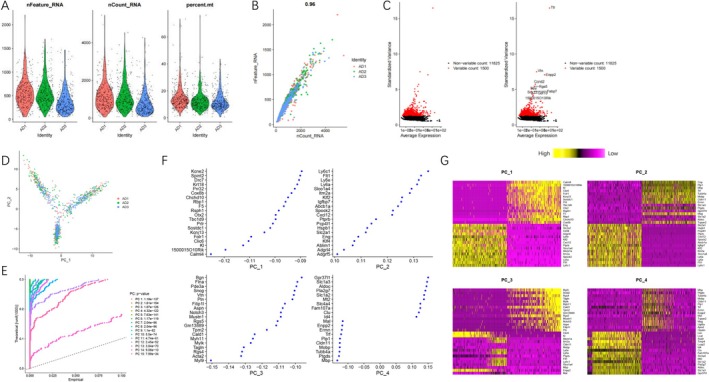
Quality control and PCA dimension reduction of scRNA‐seq data in the AD group. (A) Violin plots showing the distribution of the number of genes (nFeature_RNA), mRNA molecules (nCount_RNA), and mitochondrial gene percentage (percent.mt) in each cell of the AD group scRNA‐seq data (*N* = 3); (B) Scatter plot showing the correlation between nCount_RNA and nFeature_RNA in the filtered data (*N* = 3); (C) Variance analysis to identify the top 1500 highly variable genes in the samples (red dots), with the top 10 genes listed on the right; (D) PCA analysis results for cells from different sample sources; (E) *p*‐Values of the first 15 principal components (PCs) obtained from PCA analysis; and (F, G) heatmaps showing the expression levels of feature genes in PC_1 and PC_4 identified from PCA analysis.

To examine the mechanism of neuroinflammation, brain tissues were extracted from mice in the NC and AD groups. Subsequently, single‐cell sequencing was conducted on three samples from each group. Following quality control and standardization of scRNA‐seq data, the filtered distribution of RNA in cells is presented in Figure 1A for the AD group and Figure S2A for the NC group, respectively. Most cells exhibit nFeature_RNA < 2000, nCount_RNA < 5000, and percent.mt < 20%. The correlation coefficients between nCount and nFeature in the NC and AD groups are 0.96 (Figure 1B) and 0.94 (Figure S2B), respectively, indicating high cell quality after filtration. After filtering cells from the AD and NC groups, 11,825 and 12,649 highly variable genes were identified. Gene expression variance analysis was then conducted to select the top 1500 highly variable genes for downstream analysis, as shown in Figure 1C and Figure S2C.

To facilitate the subsequent clustering and annotation of cells, we conducted PCA on each of the groups mentioned above, thereby reducing the dimensionality of 1500 genes (Figure 1D; Figure S2D). After performing dimension reduction using PCA, we visualized the “important” principal components (PCs) using the JackStrawPlot function. We compared the distribution of *p*‐values for each PC to the distribution of the mean. The “important” PCs (solid line above the dashed line) are representative of the information contained in genes that exhibit high variability (Figure 1E; Figure S2E). In this study, we present the characteristic genes of PC_1 and PC_4 (Figure 1F; Figure S2F), accompanied by the heatmap of their expression levels shown in Figure 1G and Figure S2G.

### 3.2 Astrocyte Predominance in AD: Insights From scRNA‐Seq Analysis

**FIGURE 2:**
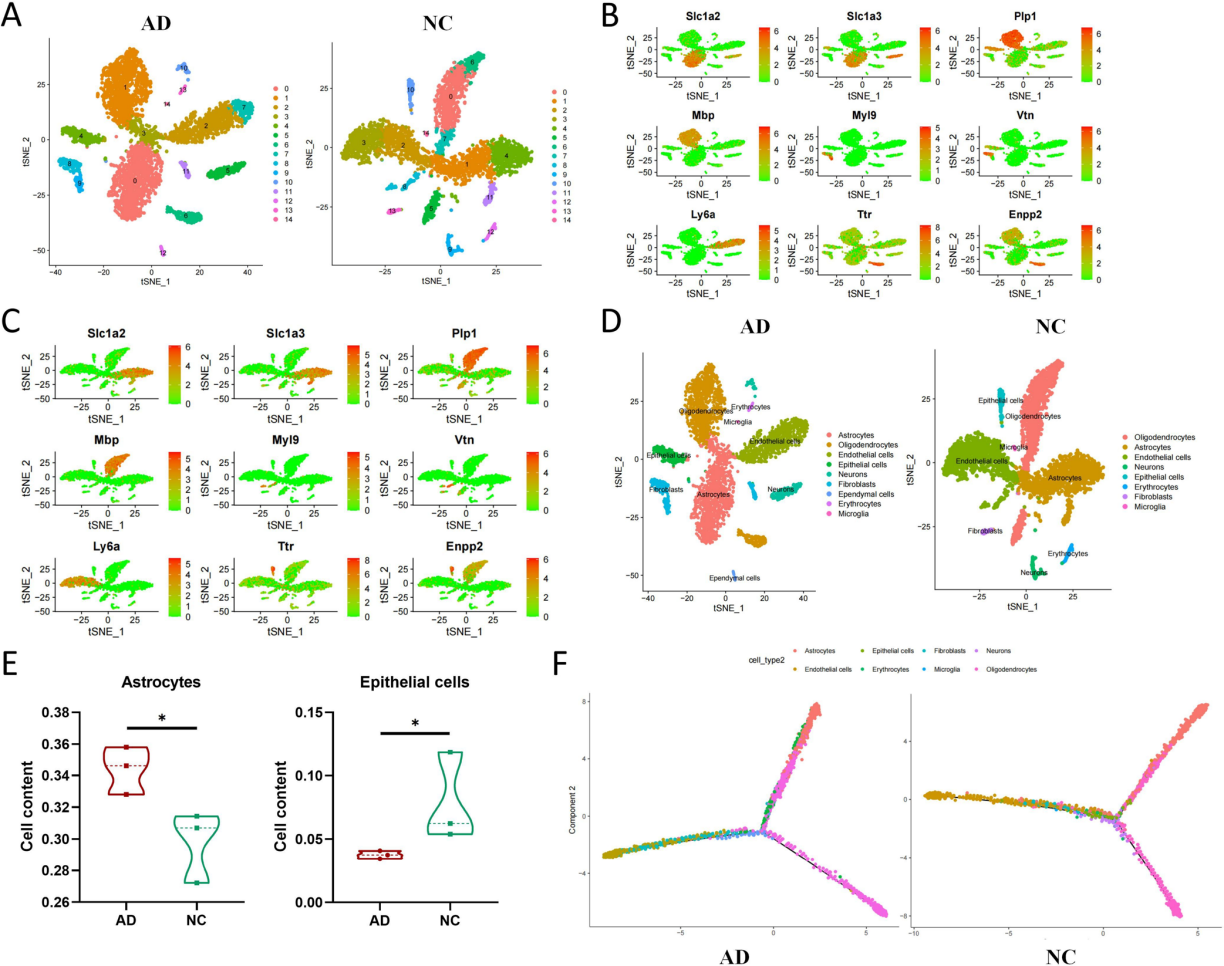
Identification of key cell types associated with AD through scRNA‐seq data analysis. (A) Visualization of TSNE clustering results showing the aggregation and distribution of cells from AD group samples (*N* = 3) and NC group samples (*N* = 3) where each color represents a cluster; (B) scatter plot showing the cell clustering of the top‐ranked marker genes in each cluster of the AD group samples; (C) scatter plot showing the cell clustering of the top‐ranked marker genes in each cluster of the NC group samples; (D) visualization of cell annotation results based on TSNE clustering, where each color represents a cell type; (E) *T*‐test results comparing the cell composition between AD group (*N* = 3) and NC group (*N* = 3), indicating differences in the occurrence of two cell types. *Represents comparison with *p*‐value < 0.05; (F) construction of cell trajectories based on cell type and clustering for pseudo‐time analysis (pseudo‐time progresses from left to right, increasing sequentially).

To explore cell types closely associated with AD, we conducted clustering analysis and cell annotation on the scRNA‐seq data using the PCA dimension reduction method mentioned earlier. The cells from the AD group and the NC group were classified into 15 clusters each, based on t‐SNE clustering analysis (Figure 2A). Marker genes and gene expression profiles for each cluster were obtained (Figure S3A). Furthermore, the expression patterns of the top‐ranking marker genes in each cluster were plotted based on the marker genes of the clusters (Figure 2B,C; Figure S3B).

Based on the marker genes of clusters, cell annotation for the clusters in the AD and NC groups was performed using the “SingleR” package in combination with the CellMarker database. In the AD group, 15 clusters were annotated into nine cell types: cluster 0 and 3 as astrocytes, cluster 2 and 7 as endothelial cells, cluster 12 as ependymal cells, cluster 4 as epithelial cells, cluster 13 as erythrocytes, cluster 8, 9, and 11 as fibroblasts, cluster 14 as microglia, cluster 5 and 10 as neurons, and cluster 1 and 6 as oligodendrocytes (Figure 2D; Table S3) [54, 55]. In the NC group, 15 clusters were annotated into eight cell types: cluster 1, 4, and 11 as astrocytes, cluster 2, 3, and 8 as endothelial cells, cluster 10 as epithelial cells, cluster 12 as erythrocytes, cluster 13 as fibroblasts, cluster 14 as microglia, cluster 9 as neurons, and cluster 0, 5, 6, and 7 as oligodendrocytes (Figure 2D; Table S4) [54, 55].

To identify the key cell types involved in AD pathogenesis, we conducted *T*‐test analyses to compare cell types between the AD and NC groups. Considering potential biases due to low cell counts of erythrocytes and microglia, we primarily compared the abundance of the other six cell types. The results indicated that astrocytes, endothelial cells, and oligodendrocytes had relatively high proportions in both groups (Table S5). Specifically, the proportion of astrocytes in the AD and NC groups was 34.41% and 30.89%, respectively, while the proportion of epithelial cells was 3.75% and 7.82%, respectively, showing a statistical difference between the AD and NC groups (Figure 2E; Figure S3C). This suggests that astrocytes may play a crucial role in AD pathogenesis. To validate this finding, we performed cell ordering and trajectory construction using the “monocle” package based on gene expression trends. The results revealed a significant increase in astrocytes during the middle‐to‐late stages of disease progression (right upper branch), with astrocytes predominating at this stage. This phase showed distinct differences in cell composition between the AD and NC groups (Figure 2F). These results suggest that astrocytes may have a role in the development of AD.

### 3.3 Trem2‐Mediated Astrocyte Transformation and Lipid Metabolism in AD: Insights From Transcriptomic Analysis

**FIGURE 3:**
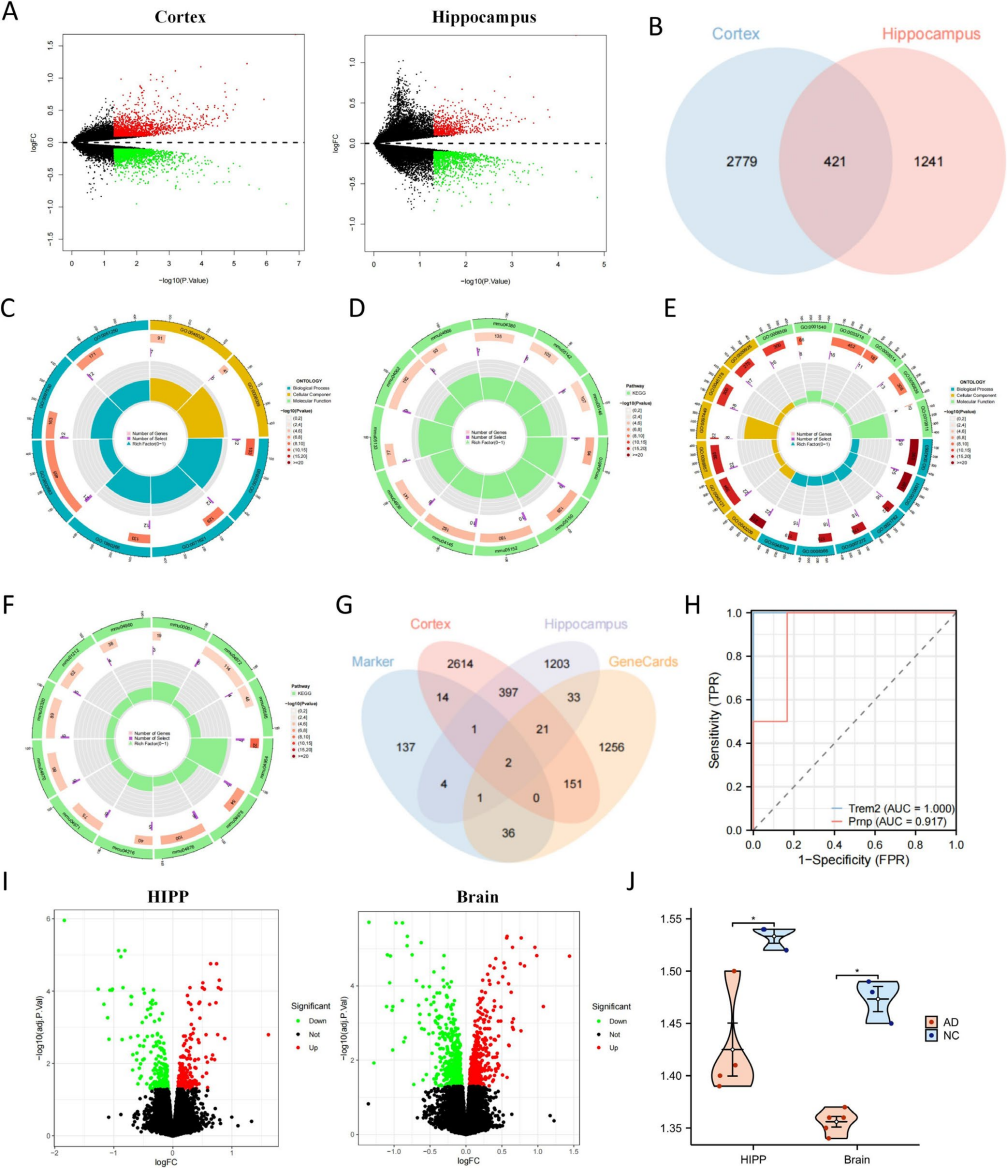
Identification of key genes involved in AD pathogenesis through transcriptome sequencing analysis. (A) volcano plot showing the differential gene expression analysis results between the AD group (*N* = 3) and NC group (*N* = 3) for the cerebral cortex and hippocampus tissues, with red indicating upregulated genes and green indicating downregulated genes; (B) Venn diagram showing the intersection of differentially expressed genes between the cerebral cortex and hippocampus tissues; (C) circle plot showing the GO functional enrichment analysis results for the intersection of differentially expressed genes; each module represents an enriched pathway; (D) circle plot showing the KEGG pathway enrichment analysis results for the intersection of differentially expressed genes; each module represents a KEGG pathway; (E) circle plot showing the GO functional enrichment analysis results for the marker genes of astrocytes derived from scRNA‐seq data; (F) circle plot showing the KEGG pathway enrichment analysis results for the marker genes of astrocytes; (G) Venn diagram showing the intersection of differentially expressed genes, marker genes, and lipid metabolic related genes between the cerebral cortex and hippocampus tissues; (H) diagnostic ROC curve for AD occurrence based on the expression of Trem2 and Prnp genes using transcriptome sequencing data; (I) Volcano plots depicting the differential expression analysis results of hippocampal tissue (HIPP) and whole brain tissue from the AD‐related dataset GSE165111 obtained from the GEO database; and (J) comparative intergroup analysis of Trem2 expression levels in the AD‐related dataset GSE165111, with * indicating comparisons between groups where *p* < 0.05.

To investigate the key genes and signaling pathways associated with astrocytes in the development of AD, we conducted transcriptome sequencing on hippocampal and cortical tissues from both the AD and control (NC) groups of mice. Subsequently, we performed a bioinformatics analysis of the obtained results. Differences were observed in the expression levels of numerous genes in AD mouse brain tissues compared to normal mouse brain tissues (Figure 3A; Figure S4A). To investigate the role of these genes in the pathogenesis of AD mediated by astrocytes, we conducted a Venn diagram analysis (Figure 3B) to identify overlapping differentially expressed genes with similar expression patterns in the two tissues. Additionally, we performed functional enrichment analysis using GO and KEGG databases for the genes common to both the differentially expressed genes and the marker genes of astrocytes.

The results of the GO functional enrichment analysis for the intersection and difference genes revealed their predominant involvement in regulating immune cells and immune responses, specifically in processes such as neutrophil chemotaxis, negative regulation of immune system processes, and antigen processing and presentation (Figure 3C; Figure S4B). Furthermore, it encompasses the regulation of neuronal morphology and function, including processes such as the positive regulation of neuronal death. Notably, these genes also participate in pathways related to lipid metabolism, such as the response to lipoprotein particles and cellular response to lipoprotein particle stimuli (Figure 3C; Figure S4B). The KEGG pathway enrichment analysis also revealed the involvement of these genes in neuroinflammation regulation, specifically the “chemokine signaling pathway” (Figure 3D; Figure S4C).

Later, a functional enrichment analysis was conducted on the marker genes of star‐shaped astrocytes. The results of the gene ontology (GO) enrichment analysis demonstrated that, in addition to multiple pathways associated with the regulation of neural function, marker genes were also found to be enriched in pathways related to lipid metabolism, specifically involved in biological functions such as “regulation of lipid metabolic process” and “positive regulation of lipid metabolic process” (Figure 3E; Figure S4D). Conversely, the KEGG enrichment analysis revealed that the majority of the pathways were primarily associated with lipid metabolism, including “bile secretion,” “fatty acid metabolism,” and “ether lipid metabolism” (Figure 3F; Figure S4E). To identify the key genes that are essential, we conducted a Venn diagram analysis on the overlap between differentially expressed genes, marker genes, and genes related to lipid metabolism obtained from the GeneCards database. This analysis resulted in the identification of two genes: Trem2 and Prnp (Figure 3G).

Furthermore, by integrating transcriptomic data, receiver operating characteristic (ROC) curves were constructed for Trem2 and Prnp to aid in diagnosis. The results demonstrated that Trem2 had superior predictive capability for AD occurrence (Figure 3H). Multiple studies have indicated a close association between Trem2 and abnormalities in lipid metabolism and neuroinflammation [56, 57]. Furthermore, research has shown that alterations in lipid metabolism levels regulate the phenotypic transformation of astrocytes, which is closely linked to the pathogenesis of AD [58].

To validate these findings, we downloaded an AD‐related transcriptome dataset, GSE165111, from the GEO database and conducted differential expression analysis on the hippocampal and brain tissue data. Applying a filtering criterion of *p* < 0.05, we identified 377 and 860 genes with differential expression between AD model mice and wild‐type mice, respectively (Figure 3I). We focused on the differential expression of Trem2 in the two tissue types and found that after removing an evident outlier, Trem2 exhibited significantly reduced expression levels in the hippocampal and brain tissues of AD mice (Figure 3J). This outcome further confirms the accuracy of our earlier results.

Based on the results above, we propose a hypothesis that Trem2 enhances neuroinflammation and plays a role in the pathogenesis of AD through its regulation of lipid metabolism and transformation of astrocytes into a pro‐inflammatory phenotype.

### 3.4 Lipid Metabolism Disruption in AD: A Metabolomic Study Revealing the Role of Trem2

**FIGURE 4:**
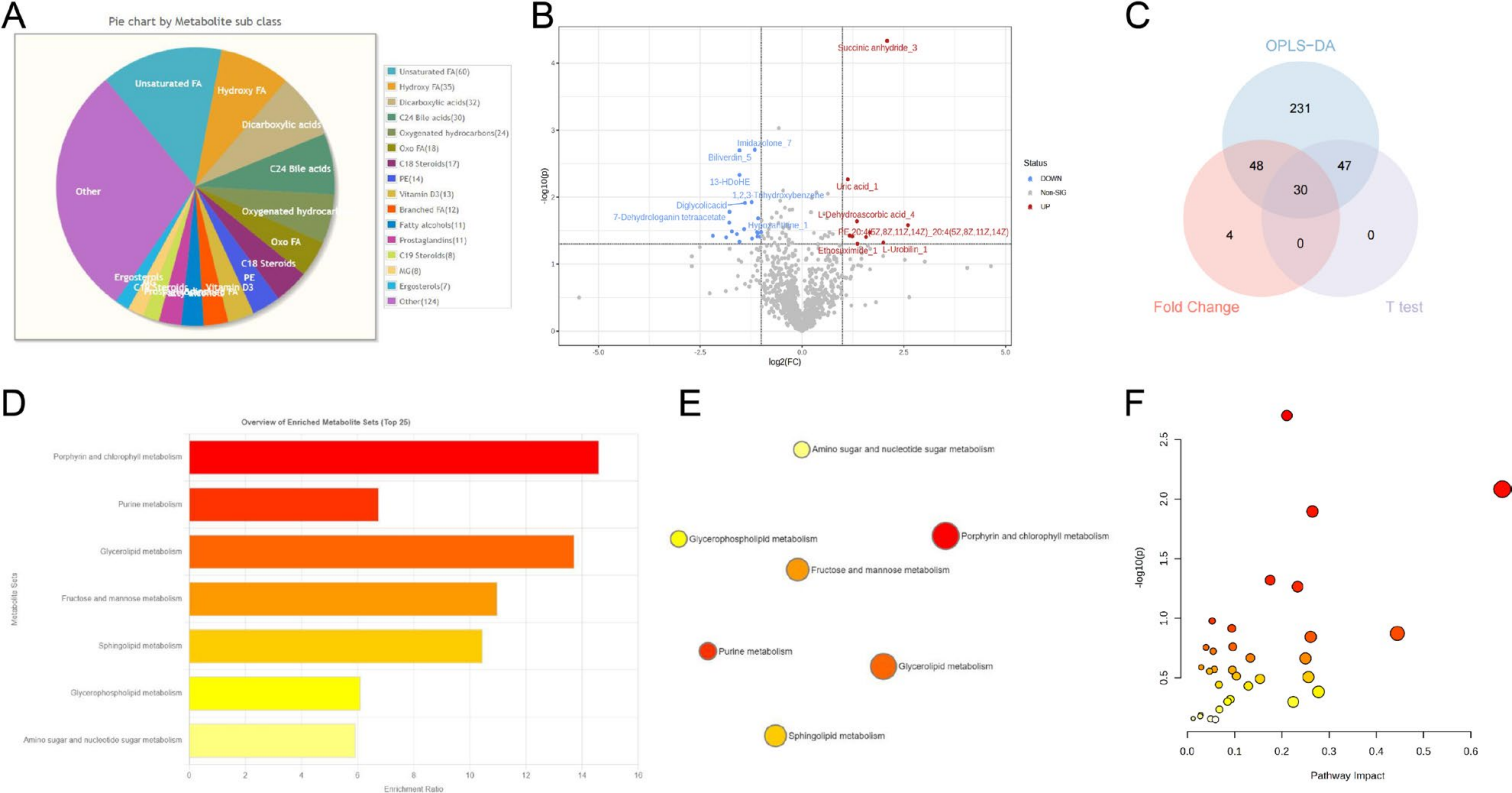
Analysis of metabolomic data to explore the relationship between lipid metabolism and AD. (A) Pie chart showing the composition of lipid‐related “Sub class” metabolites; (B) volcano plot showing the differential metabolites analyzed based on fold change and *T*‐test; (C) Venn diagram showing the intersection of differential metabolites obtained by OPLS‐DA analysis with VIP > 1, fold change analysis |logFC| > 2, and *T*‐test *p* < 0.05; (D) bar plot showing the functional enrichment analysis results of differential metabolites in the MetaboAnalyst database; (E) network plot showing the functional enrichment analysis results of differential metabolites in the MetaboAnalyst database; (F) comprehensive metabolic pathway analysis of differential metabolites combined with astrocytes in the MetaboAnalyst database. Each group of samples has *N* = 15.

To validate the impact of lipid metabolism on the occurrence of AD based on the bioinformatics above analysis, we performed a comprehensive analysis of metabolites in brain tissues from both normal mice and AD mice using high‐throughput metabolomics techniques. To streamline subsequent statistical analysis, we annotated and complemented missing metabolite data. This result was followed by data calibration and normalization (Figure S5A). Subsequently, we analyzed the composition of metabolites in the samples. Based on the macro classification of metabolites into “Super class” and the more detailed classification “Main class,” we found that fatty acid metabolites accounted for a higher proportion among all metabolites (Figure S5B). According to the classification of lipid‐related metabolites under the “Subclass” category, it was observed that unsaturated fatty acids and hydroxy fatty acids had the highest proportion (Figure 4A). To identify metabolic products closely associated with AD, we performed orthogonal partial least squares discriminant analysis (OPLS‐DA) on the corrected data. This method effectively minimizes intra‐group variation and random errors unrelated to the research objective while also preventing overfitting. The score plot demonstrates the model's ability to distinguish between samples from the AD and NC groups (Figure S5C).

We selected metabolites for further analysis based on their variable importance in projection (VIP) scores from the OPLS‐DA model (Figure S5D). A threshold of VIP > 1 was applied, and the top 15 metabolites with the highest VIP values are presented in Figure S5E. Additionally, 30 differential metabolites were identified using fold change analysis (|logFC| > 2; Figure S5F) and a *t*‐test (*p* < 0.05; Figure S5G). These metabolites, which intersect the criteria of VIP > 1, |logFC| > 2, and *p* < 0.05, are shown in Figure 4C, highlighting their association with AD.

Metabolites from the differential metabolism were uploaded to the MetaboAnalyst website for pathway enrichment analysis. The analysis revealed several pathways, including “glycerolipid metabolism” and “sphingolipid metabolism,” that were linked to lipid metabolism. These findings are depicted in Figure 4D,E and Figure S6A. The KEGG pathway diagram for “sphingolipid metabolism” is provided in Figure S6B. Finally, we conducted a comprehensive metabolic pathway analysis to elucidate the relationship between astrocyte cells and lipid metabolism. This analysis involved combining astrocyte cell marker genes with differential metabolites. The results indicate that there was a notable enrichment of marker genes and differential metabolites mainly in metabolic pathways such as ether lipid metabolism, glycerolipid metabolism, sphingolipid metabolism, and fatty acid degradation, as depicted in Figure 4F and Figure S6C–F. Previous studies have reported that Trem2 could regulate lipid metabolism processes, such as phospholipid and fatty acid metabolism and participate in developing AD [58, 59].

Based on the analysis above, it is hypothesized that Trem2 influences the occurrence of neuroinflammation linked to AD by modulating lipid metabolism processes, including phospholipid metabolism and fatty acid degradation.

### 3.5 Modulation of Astrocyte‐Mediated Inflammation by Trem2 in a Cellular Model of Neuroinflammation

To validate the findings from the analysis as mentioned above, primary astrocytes were isolated and cultured from mice in the control (NC) group. These cells were then stimulated with LPS to mimic an inflammatory response and subjected to relevant tests. We observed the cell morphology through an inverted microscope and found that starting from the third day, irregular polygons with multiple projections appeared on the cell body. By the 11th day, the cells had completely covered the entire culture dish, which aligns with the growth characteristics typically observed in astrocytes (Figure S7A). Utilizing GFAP immunofluorescent staining, we observed red fluorescence in the cytoplasm of cells, indicating the presence of filamentous material produced by the glial fibrillary acidic protein (GFAP).

**FIGURE 5:**
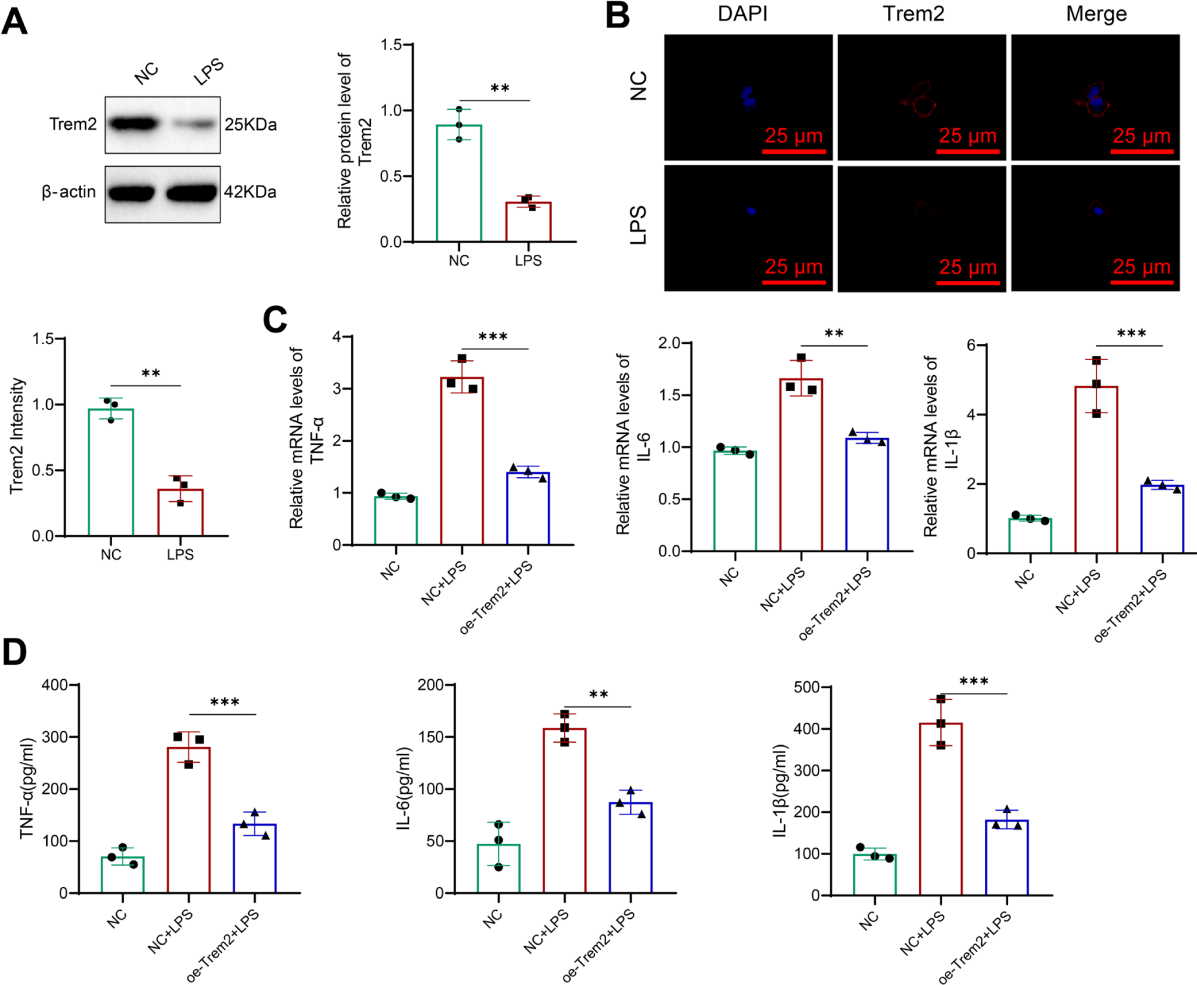
Relationship between Trem2 and LPS‐induced neuroinflammation. (A) Western blot analysis of Trem2 protein expression in astrocytes after LPS stimulation; (B) immunofluorescence staining and fluorescence intensity analysis of Trem2 in astrocytes after LPS stimulation; (C) RT‐qPCR analysis of TNF‐α, IL‐6, and IL‐1β expression changes in astrocytes overexpressing Trem2; and (D) ELISA measurement of TNF‐α, IL‐6, and IL‐1β levels. **p < 0.01, ***p < 0.001, respectively, with *N* = 3 in all experiments. NC (normal control): cells from non‐treated, normal astrocytes; LPS (lipopolysaccharide): astrocytes stimulated with LPS to induce inflammation as a model of neuroinflammation; Oe‐Trem2 (overexpressed Trem2): astrocytes that were transfected with Trem2 to study its effect in modulating the LPS‐induced inflammatory response.

Additionally, the cell nucleus displayed blue fluorescence, a characteristic feature of GFAP staining in astrocytes. The positivity rate exceeds 95% and is suitable for subsequent experiments (Figure S7B). We assessed the impact of LPS on Trem2 expression levels using western blot and immunofluorescence assays. The results demonstrated a decrease in the expression level of Trem2 following LPS stimulation (Figure 5A,B).

To evaluate the impact of Trem2 on the inflammation response mediated by astrocytes, we applied an overexpression of Trem2 (Figure S7C). The overexpression of Trem2 in astrocytes was confirmed through RT‐qPCR and western Blot analysis (Figure S7D,E). Following this, we stimulated astrocytes that were either transfected or untransfected with LPS and evaluated the expression levels of pertinent pro‐inflammatory factors using RT‐qPCR and ELISA. The results demonstrated that overexpression of Trem2 led to decreased levels of mRNA expression of TNF‐α, IL‐6, and IL‐1β in activated microglial cells following LPS stimulation, as illustrated in Figure 5C,D.

Therefore, in conclusion, Trem2 hampers the inflammatory response mediated by astrocytes.

### 3.6 Inhibition of NF‐κB Activation by Trem2 Mitigates Inflammatory Response in Astrocytes

TLR4 is a key receptor for LPS signaling. Studies have shown that Trem2 can inhibit TLR4‐mediated NF‐κB activation in Parkinson's disease [44]. Inactive NF‐κB predominantly exists as a p65‐p50 dimer localized in the cytoplasm. When NF‐κB is activated, the p65 subunit is released and translocated into the nucleus via a nuclear localization signal to initiate transcriptional activation of downstream genes [60, 61, 62]. However, the role of Trem2 in astrocyte‐mediated neuroinflammation and the progression of AD remains uncertain. Based on previous bioinformatic analyses and literature reports, we hypothesized that low Trem2 expression may promote TLR4‐mediated NF‐κB activation and the production of inflammatory cytokines in astrocytes.

**FIGURE 6:**
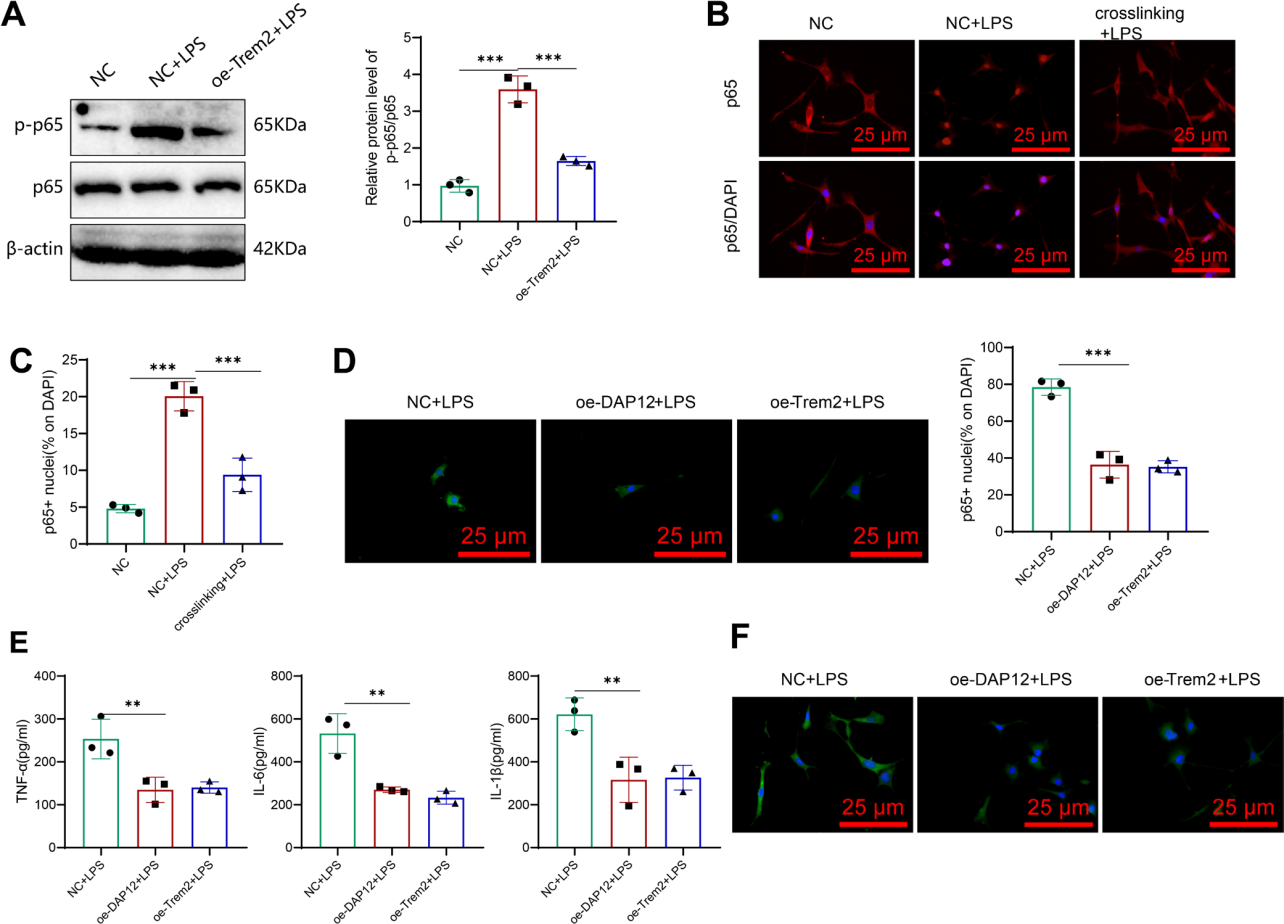
Trem2 modulates the production of inflammatory factors by regulating NF‐κB activation. (A) Expression and activation levels of the key downstream signaling pathway NF‐κB subunit p65 were detected by western blot after TLR4 activation; (B) representative images of immunofluorescent staining of nuclear p65 subunit localization after Trem2 antibody cross‐linking; (C) quantitative results of nuclear p65 subunit localization; (D) representative images and quantitative results of fluorescent staining of nuclear p65 subunit localization after Trem2 or DAP12 transfection; (E) ELISA measured levels of TNF‐α, IL‐6, and IL‐1β; and (F) immunofluorescent staining was performed to observe morphological changes in transfected cells. **p < 0.01, ***p < 0.001, respectively. *N* = 3.

To test this hypothesis, we measured the levels of p65 and other key downstream signaling molecules activated by TLR4, such as ERK and JNK. The results showed that, after LPS stimulation, the phosphorylation level of the NF‐κB subunit p65 was significantly increased in cells compared to the NC group. However, in Trem2‐overexpressing astrocytes, LPS‐induced phosphorylation of p65 was significantly reduced (Figure 6A), while the phosphorylation of ERK and JNK was unaffected (Figure S8A,B). These findings suggest that the reduced expression of pro‐inflammatory cytokines in Trem2‐overexpressing astrocytes may result from decreased activation of the NF‐κB signaling pathway.

To further validate this, we used Trem2‐specific antibodies for crosslinking to mimic Trem2 activation, as described in the literature [44], and quantified the nuclear localization of the p65 subunit. The results showed that Trem2 crosslinking inhibited LPS‐induced NF‐κB activation in astrocytes (Figure 6B,C). DAP12 is a pairing partner protein of Trem2, and upon Trem2 activation, DAP12 is subsequently activated, triggering a cascade of intracellular signaling events [63]. Given the potential non‐specific binding issues associated with antibody crosslinking, we transfected astrocytes with Trem2 and DAP12 (Figure S8C), followed by LPS stimulation, and quantified the nuclear localization of the NF‐κB subunit p65 and the levels of pro‐inflammatory cytokines. The results showed that, compared to the control group, Trem2‐ or DAP12‐transfected astrocytes exhibited reduced NF‐κB activation (Figure 6D) and decreased levels of inflammatory cytokines such as TNF‐α, IL‐6, and IL‐1β after LPS stimulation (Figure 6E). Furthermore, the cells predominantly displayed a polygonal morphology rather than the reactive astrocyte phenotype (Figure 6F). These findings further confirm that Trem2 overexpression regulates the production of inflammatory cytokines and the transition to a pro‐inflammatory phenotype by inhibiting NF‐κB activation.

### 3.7 Deciphering the Impact of Trem2 on Astrocyte Lipid Metabolism: A Lipidomic Analysis Post‐LPS Stimulation

**FIGURE 7:**
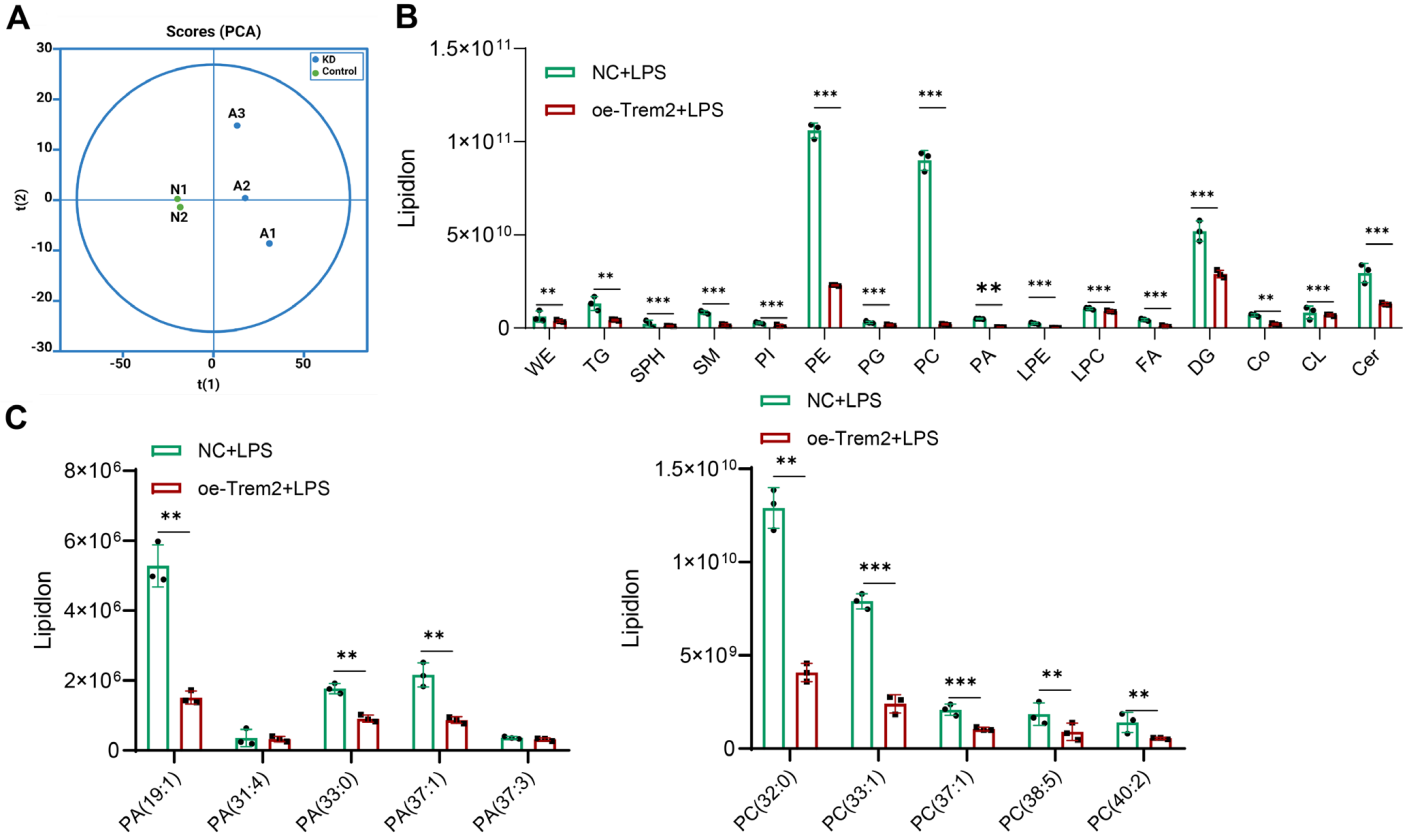
Lipidomics study of the effect of Trem2 on lipid metabolism in astrocytes. (A) PCA score plot of non‐targeted lipidomic data. (B) Lipid species with differences (*p* < 0.05, VIP > 1) between the NC group and oe‐Trem2 group after LPS stimulation. (C) Differences in the levels of certain lipid subclasses, such as PC and PA. **p < 0.01, ***p < 0.001, respectively. *N* = 3.

Previous studies have demonstrated that alterations in lipid metabolism modulate the production of inflammatory mediators [56, 57, 64]. Our previous metabolomic analysis demonstrated that lipid metabolism is crucial in developing astrocyte‐associated neuroinflammation. We experimented to investigate the impact of Trem2 on lipid metabolism in cells after LPS stimulation. Additionally, we aimed to confirm the specific types of lipids associated with Trem2‐mediated astrocyte pro‐inflammatory phenotype transition. To achieve this, we employed untargeted lipidomic sequencing to identify alterations in lipid composition in both Trem2‐overexpressing cells and cells stimulated with LPS. PCA was used to analyze non‐targeted lipidomic data, which confirmed the reliability of the sequencing data (Figure 7A).

Using *T*‐test and OPLS‐DA revealed changes in the levels of lipid components in astrocytes after overexpression of Trem2 (*p* < 0.05, VIP > 1). This result includes a decrease in phospholipids (PE, PC, PA, PI, and PG), sphingolipids (Ger, SM), and fatty acids (FA) (Figure 7B). Furthermore, we detected distinct variations in the levels of specific lipid subclasses, including phosphatidylcholine (PCs) and phosphatidic acid (PAs), among others (Figure 7C). These findings provide additional evidence of Trem2's potential role in regulating inflammation through its impact on the lipid composition of astrocytes.

### 3.8 Elucidating the Role of Trem2 in Neuroinflammation and Cognitive Decline in AD

**FIGURE 8:**
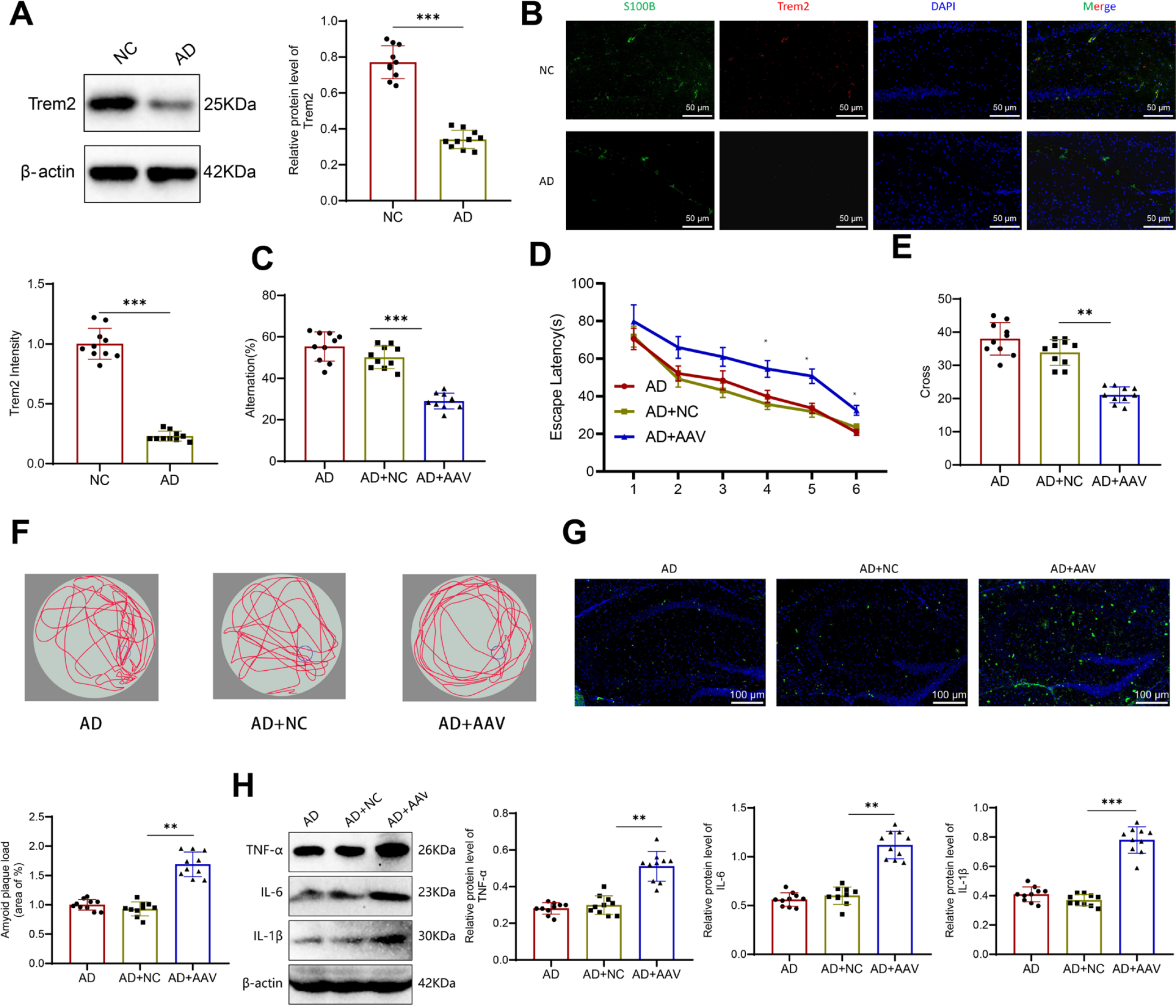
In vivo experiments validate the impact of Trem2 on neuroinflammation and AD development. (A) western blot was performed to measure the protein expression level of Trem2 in the mouse hippocampal region. (B) Immunofluorescent staining and analysis of the fluorescent intensity of S100B (green) and Trem2 (red) co‐staining in the mouse hippocampal region. (C) Spontaneous alternation rate of mice in the Y‐maze task. (D) Latency in the water maze task. (E) Number of platform crossings in the water maze task. (F) Movement traces of mice in the water maze task. (G) Quantification and representative imagesof plaque count in the mouse hippocampal DG region using thioflavin‐S staining. (H) Western blot was performed to measure the expression levels of inflammatory factors TNF‐α, IL‐6, and IL‐1β in the mouse hippocampal region. **p < 0.01, ***p < 0.001, respectively. *N* = 10.

To explore the role of Trem2 in neuroinflammation and AD, we proceeded to validate our hypothesis. To begin, we compared Trem2 protein expression in the hippocampus of mice from both the NC and AD groups, utilizing western blot and immunofluorescence staining techniques. The results indicate a reduction in the expression level of Trem2 protein in the hippocampal region of mice belonging to the AD group (Figure 8A). Immunofluorescence staining demonstrated a notable decrease in the expression of Trem2 protein in the hippocampal region of AD mice. This reduction was visually confirmed through colabeling with the astrocyte marker S100B (Figure 8B). These results further validate the link between Trem2 and the advancement of AD facilitated by astrocytes.

Following this, we administered a brain stereotactic injection of adenovirus into the dentate gyrus (DG) region of mice to disrupt the expression of the Trem2 gene. It is illustrated in Figure S9A. The mice were divided into three groups: the AD group, the AD + NC group, and the AD + AAV group. The results from RT‐qPCR and western Blot experiments demonstrated a successful knockdown of Trem2 expression (Figure S9B,C). Subsequently, we performed behavioral assessments related to AD on each group of mice. Open‐field experiments revealed no differences in the total distance and central time among the three groups of mice, indicating that Trem2 does not impact mouse anxiety‐like behavior (Figure S9D). In the assessment of memory and cognitive function through the Y‐maze test, the AD + AAV group of mice exhibited a significantly lower rate of spontaneous alternation compared to the other two groups (Figure 8C). In order to further examine the impact of Trem2 on the cognitive function and memory of AD mice, we employed the Morris water maze test. The findings demonstrated no noteworthy disparity in the average swim speed during the training phase among the three mouse groups (Figure S9E). Nevertheless, as the training advanced, the AD + AAV mouse group displayed a considerably longer latency period in comparison to the other two groups (Figure 8D) and exhibited a reduction in the number of times they crossed the hidden platform (Figure 8E,F). These findings suggest that the reduction of Trem2 worsens cognitive impairment in mice.

Finally, we conducted an additional investigation to examine the influence of Trem2 on the formation of amyloid plaques and inflammatory factors in the hippocampus of mice. Sulfur compounds stained the hippocampal region in three groups of mice, and the AD + AAV group exhibited an increase in amyloid burden in aging mice (Figure 8G). The results of protein imprinting reveal increases in the expression levels of inflammatory factors TNF‐α, IL‐6, and IL‐1β in the hippocampus of mice in the AD + AAV group (Figure 8H). These findings provide additional support for our hypothesis that Trem2 plays a pivotal role in regulating neuroinflammation through modulation of microglial pro‐inflammatory transformation, which contributes to the onset and progression of AD.

## 4 Discussion

In this study, we performed single‐cell sequencing on brain tissues from a mouse model of AD. We found an increase in the abundance of astrocytes, along with the upregulation of specific markers. These findings align with previous studies indicating the involvement of astrocytes in the pathogenesis of AD. Moreover, they provide further insights into this field [15, 16, 65]. Previous studies have primarily concentrated on the neuroprotective and inflammatory roles of astrocytes. In contrast, our study delves deeper into their contribution to lipid metabolism, offering a fresh perspective for AD research [66].

Astrocytes are integral to the central nervous system, and emerging evidence suggests that abnormalities in lipid metabolism within these cells can contribute to the phenotypic transformation of astrocytes, which is particularly relevant in AD [67, 68]. This transformation is characterized by an increased inflammatory response and altered functional properties of astrocytes [69, 70]. In AD, lipid dysregulation within astrocytes can lead to a shift from a homeostatic to a reactive phenotype, exacerbating neuroinflammation. Our data support the notion that the modulation of lipid metabolism may not only influence the inflammatory status of astrocytes but also their phenotypic transition, thus playing a critical role in AD progression. These findings further highlight the potential of targeting lipid metabolic pathways in astrocytes as a therapeutic strategy to mitigate neuroinflammation and its contribution to AD pathology.

The expression of Trem2 is not confined to microglial cells but is also found in other cell types, including astrocytes and neurons [25]. This broad expression suggests that Trem2 may play a role in various biological processes, such as immune regulation, cell proliferation, and survival. Recent studies indicate that Trem2 expression in astrocytes may be linked to the pathological processes in neurodegenerative diseases, including AD [27]. Our research further highlights the critical role of Trem2 in astrocytes during AD progression.

Our findings show significant alterations in lipid metabolism in astrocytes during AD progression, challenging the traditional view that AD pathology is primarily driven by the abnormal accumulation of β‐amyloid and tau proteins. Instead, our data present lipid metabolism as a key player in AD [71]. Although lipid metabolism changes have been observed in other neurodegenerative diseases, research in AD has been limited [72, 73, 74, 75].

Using transcriptome sequencing of AD mouse model brain tissues, we identified several key genes that may regulate lipid metabolism. The 5xFAD mouse model effectively mimics AD characteristics [76], and our reliable single‐cell sequencing analysis supports its use for cell clustering and annotation. These genes, though previously underexplored, may play significant roles in AD pathogenesis. Further investigation could reveal new therapeutic targets. In addition, we employed high‐throughput metabolomics to study AD at the metabolic level, offering a novel approach to understanding the disease's etiology and allowing us to directly observe changes in cellular function. Although metabolomics in AD research is still in its early stages, it has already made significant contributions in other areas, such as cancer and cardiovascular diseases [77].

Our study suggests that lipid metabolism alterations in astrocytes could influence neuroinflammation and potentially accelerate AD progression. While neuroinflammation has long been recognized as a key factor in AD pathogenesis, its underlying mechanisms remain unclear [78]. We propose that lipid metabolism may be the link connecting astrocytes and neuroinflammation in AD.

In summary, our research presents a new perspective on AD pathogenesis, highlighting the role of astrocyte lipid metabolism and Trem2 regulation in influencing neuroinflammation and AD progression. Although our findings are promising, further validation and exploration of the detailed mechanisms are needed. Regulating lipid metabolism or Trem2 activity could offer new avenues for AD therapy. We anticipate that future studies will build on our findings and develop novel strategies for AD treatment.

Despite the important findings of this study, there are some limitations. Although our research proposes new hypotheses, these need validation in larger sample sizes and other commonly used AD models due to limitations in the animal model, pathological state, sample collection, and experimental conditions. Since our study primarily relies on animal models, clinical verification is necessary to determine its applicability to humans. Our results suggest that lipid metabolism in astrocytes may influence AD pathogenesis, but the precise mechanisms remain unclear. For instance, what factors drive lipid metabolism changes in astrocytes? How do these changes impact neuroinflammation? These questions require further investigation.

Future research should explore strategies to regulate lipid metabolism in astrocytes or modulate Trem2 activity to influence lipid metabolism. We also aim to better understand the relationship between lipid metabolism alterations and neuroinflammation in AD development.

## Author Contributions

Chenhui Zhao designed and performed the majority of the experiments, including single‐cell RNA sequencing, transcriptomics, and metabolomics analyses and drafted the manuscript. Wei Qi assisted with experimental design, conducted in vitro and in vivo studies, and contributed to data analysis. Xiaoping Lv and Xueli Gao supported data interpretation and provided critical revisions to the manuscript. Chaonan Liu performed additional statistical analyses and helped with figure preparation. Shimin Zheng supervised the study, provided funding, and critically reviewed the manuscript. All authors read and approved the final manuscript.

## Ethics Statement

This study was approved by the Animal Ethics Committee of the Harbin Veterinary Research Institute of the Chinese Academy of Agricultural Sciences. All the procedures were in accordance with the guidelines on the Animal Care and Use Committee of Heilongjiang Province (SYXK (Hei) 2012–2067).

## Conflicts of Interest

The authors declare no conflicts of interest.

## Supporting information


**Figure S1.** Evaluation of AD characteristics in 5xFAD mice. (A) trend chart showing the changes in escape latency during the Morris water maze behavioral test in AD and NC mice repeated over 5 days (*N* = 10); (B) route map of AD and NC mice in the spatial exploration test (red circle denotes hidden platform), with a statistical bar chart on the right showing the number of times the mice crossed the hidden platform (*N* = 10); (C) thioflavin‐S staining quantifying the number of senile plaques (green) in the DG region of the mouse hippocampus (*N* = 10); and (D) serum Nfl concentration detected using single‐molecule array technology (*N* = 10). ***p < 0.001, for comparison between the two groups.

**Figure S2.** Quality control and PCA dimensionality reduction of scRNA‐seq data in the NC group. (A) Violin plots depicting the gene number per cell (nFeature_RNA), mRNA molecule count (nCount_RNA), and percentage of mitochondrial genes (percent.mt) in each cell of the NC scRNA‐seq data (*N* = 3); (B) scatter plot showing the correlation between filtered data nCount_RNA and nFeature_RNA (*N* = 3); (C) 1500 highly variable genes (red dots) selected by variance analysis in the samples, with the top 10 genes ranked and labeled on the right; (D) PCA results of cells from different sample sources; (E) *p*‐values of the top 15 PCs obtained from PCA analysis; and (F, G) heatmaps showing the expression levels of feature genes and their corresponding expression in PC_1 and PC_4 in the PCA analysis.

**Figure S3.** Analysis of cell clustering results in scRNA‐seq data. (A) heatmap displaying the top 10 marker gene expressions in each cluster; (B) bubble plot showing the expression pattern of selected marker genes in each cluster; and (C) *T*‐test results indicating no statistical difference in the cell type between the AD group (*N* = 3) and NC group (*N* = 3).

**Figure S4.** Differential analysis and functional enrichment analysis of transcriptome sequencing results. (A) circular heatmap showing the differential expression profiles of the top 100 genes with the most differential expression in the transcriptome sequencing results of the cerebral cortex and hippocampal tissues in the AD group (*N* = 3) compared to the NC group (*N* = 3); (B) bar plot of GO functional enrichment analysis for the intersection of differentially expressed genes, with the top five pathways ranked in biological processes (BP), cellular components (CC), and molecular functions (MF); (C) bar plot of KEGG pathway enrichment analysis for the intersection of differentially expressed genes; (D) bar plot of GO functional enrichment analysis for marker genes of astrocytes; and (E) bar plot of KEGG pathway enrichment analysis for marker genes of astrocytes.

**Figure S5.** Quality control and multivariate statistical analysis of metabolomic data. (A) comparison of raw metabolomics data before and after correction and normalization; (B) pie charts showing the composition of metabolites categorized based on “Super class” and “Main class”; (C) score plot of OPLS‐DA analysis, with the horizontal and vertical axes representing the scores of the primary component and orthogonal component, respectively, representing inter‐group and intra‐group differences; (D) S‐plot of OPLS‐DA analysis, with metabolites closer to the corners indicating higher importance in the model; (E) VIP values of top‐ranking metabolites based on OPLS‐DA analysis; (F) score plot of fold change analysis, with red dots representing |logFC| > 2 in comparisons between the two groups; and (G) *T*‐test analysis results, with red dots representing *p* < 0.05 in comparisons between the two groups. Each group contains *N* = 6 samples.

**Figure S6.** Functional and pathway enrichment analysis of differential metabolites. (A) bubble plot of functional enrichment analysis results of differential metabolites in the MetaboAnalyst database; (B) pathway enrichment analysis results of differential metabolites in the MetaboAnalyst database, with the display of the “Sphingolipid metabolism” pathway and enriched metabolites on the right; and (C–F) the “Ether lipid metabolism,” “Glycerolipid metabolism,” “Sphingolipid metabolism,” and “Fatty acid degradation” pathways in the integrated metabolic pathway analysis results.

**Figure S7.** Identification of astrocytes and transfection assessment. (A) Changes in growth conditions of primary astrocytes observed under an inverted microscope on days 1, 3, 5, 7, 9, and 11; (B) immunofluorescence staining of specific astrocytic marker GFAP; (C) observation of Trem2 overexpression plasmid transfection effect under a microscope; (D) expression level of Trem2 in transfected cells detected by RT‐qPCR; and (E) expression level of Trem2 protein in transfected cells detected by western Blot. **p < 0.01, ***p < 0.001 between the two groups, *N* = 3. NC (normal control): cells from non‐treated, normal astrocytes; Oe‐Trem2 (overexpressed Trem2): astrocytes that were transfected to overexpress Trem2 to study the effects of Trem2 overexpression on inflammation.

**Figure S8.** Mechanistic study of Trem2’s impact on inflammatory factor production. (A) Expression and activation levels of key downstream signaling molecule ERK in TLR4‐activated cells detected by western Blot; (B) expression and activation levels of key downstream signaling molecule JNK in TLR4‐activated cells detected by western Blot; and (C) observation of Trem2 and DAP12 overexpression plasmid transfection effect under a microscope. NS represents no statistical difference between the two groups, *N* = 3.

**Figure S9.** Effects of Trem2 knockdown on mouse AD behavior. (A) Schematic representation of stereotaxic injection of Trem2 shRNA into the mouse brain to interfere with Trem2 expression in AD mice; (B) mRNA expression level of Trem2 in the mouse hippocampus detected by RT‐qPCR; (C) protein expression level of Trem2 in the mouse hippocampus detected by western Blot; (D) time spent in the center area and total distance traveled by mice in the open field test; and (E) swimming speed of mice during the 6‐day water maze test. *p < 0.05 between the two groups, *N* = 10.

**Table S1.** Antibody information.
**Table S2.** Primer sequence.
**Table S3.** Clustering analysis and cell annotation results of AD group.
**Table S4.** Clustering analysis and cell annotation results of NC group.
**Table S5.** Proportion of different types of cells in AD and NC groups.

## Data Availability

The data that support the findings of this study are available from the corresponding author upon reasonable request.
