# Supplementary figures and images for: Elucidating the Role of Trem2 in Lipid Metabolism and Neuroinflammation
Source: CNS Neurosci Ther. 2025 Apr 9;31(4):e70338. doi: 10.1111/cns.70338 (PMC11982525; doi:10.1111/cns.70338)

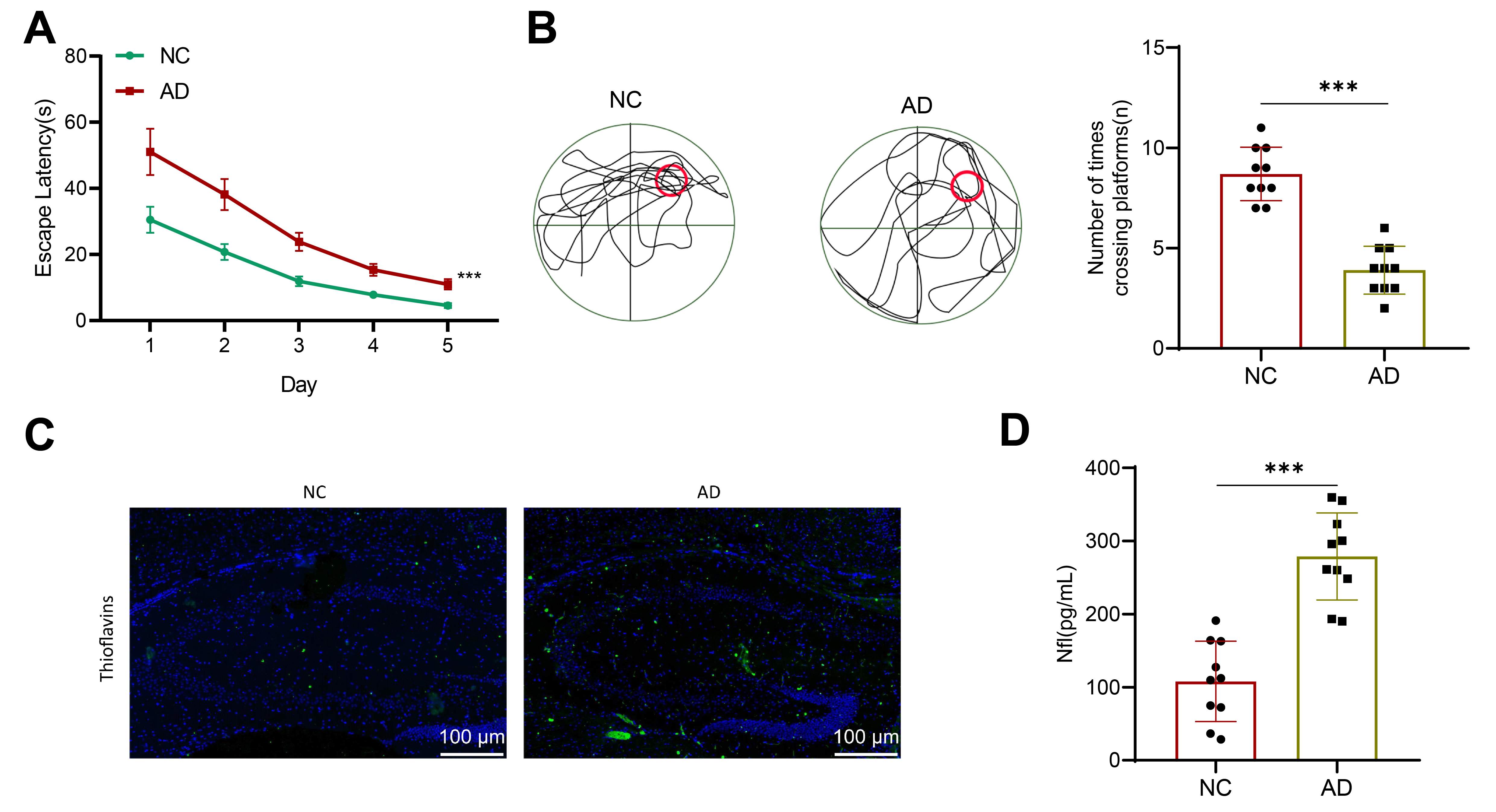

Supplement: Supplementary file 1 — Figure S1. Evaluation of AD characteristics in 5xFAD mice. (A) trend chart showing the changes in escape latency during the Morris water maze behavioral test in AD and NC mice repeated over 5 days (N = 10); (B) route map of AD and NC mice in the spatial exploration test (red circle denotes hidden platform), with a statistical bar chart on the right showing the number of times the mice crossed the hidden platform (N = 10); (C) thioflavin‐S staining quantifying the number of senile plaques (green) in the DG region of the mouse hippocampus (N = 10); and (D) serum Nfl concentration detected using single‐molecule array technology (N = 10). ***p < 0.001, for comparison between the two groups. [file CNS-31-e70338-s008.jpg]

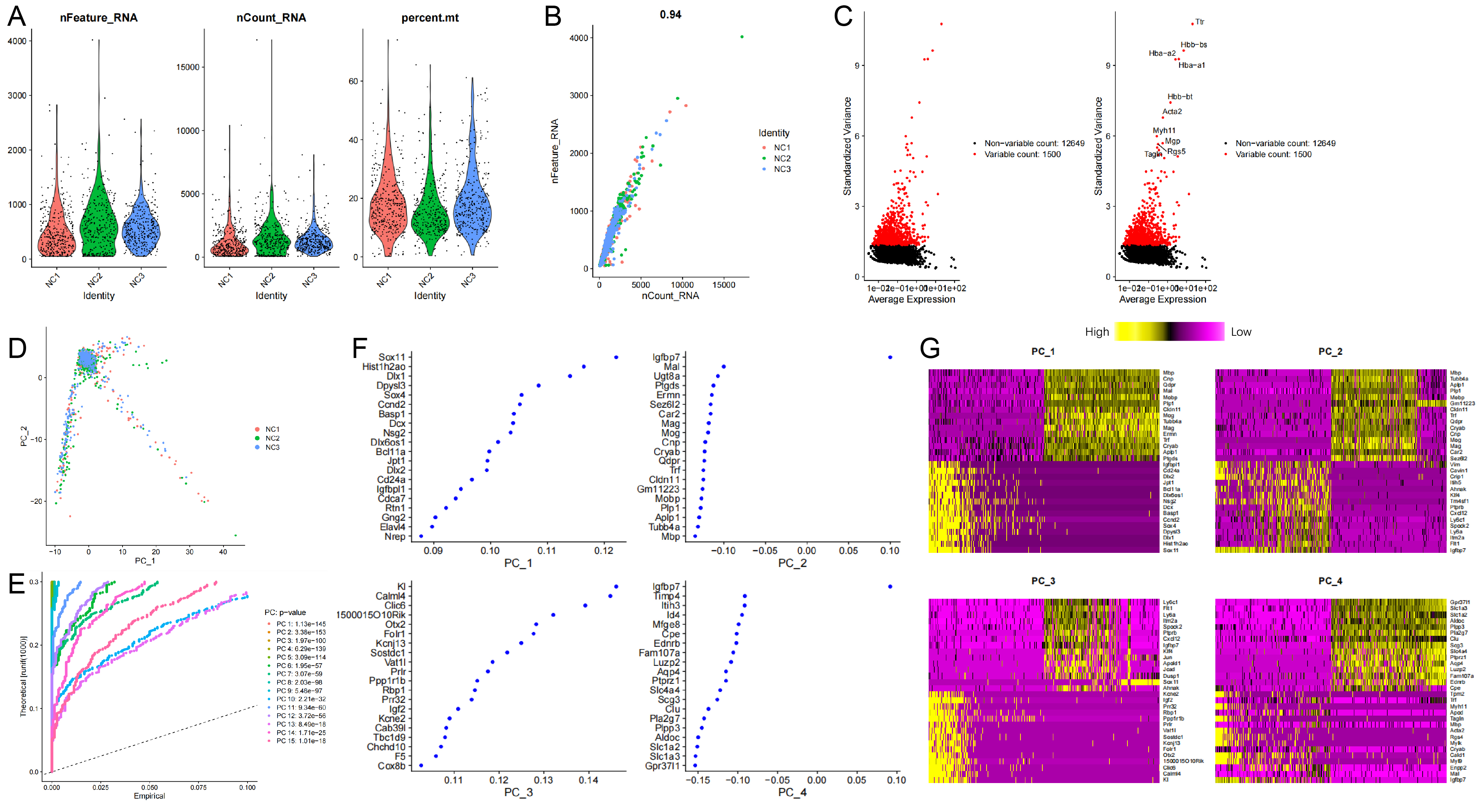

Supplement: Supplementary file 2 — Figure S2. Quality control and PCA dimensionality reduction of scRNA‐seq data in the NC group. (A) Violin plots depicting the gene number per cell (nFeature_RNA), mRNA molecule count (nCount_RNA), and percentage of mitochondrial genes (percent.mt) in each cell of the NC scRNA‐seq data (N = 3); (B) scatter plot showing the correlation between filtered data nCount_RNA and nFeature_RNA (N = 3); (C) 1500 highly variable genes (red dots) selected by variance analysis in the samples, with the top 10 genes ranked and labeled on the right; (D) PCA results of cells from different sample sources; (E) p‐values of the top 15 PCs obtained from PCA analysis; and (F, G) heatmaps showing the expression levels of feature genes and their corresponding expression in PC_1 and PC_4 in the PCA analysis. [file CNS-31-e70338-s006.jpg]

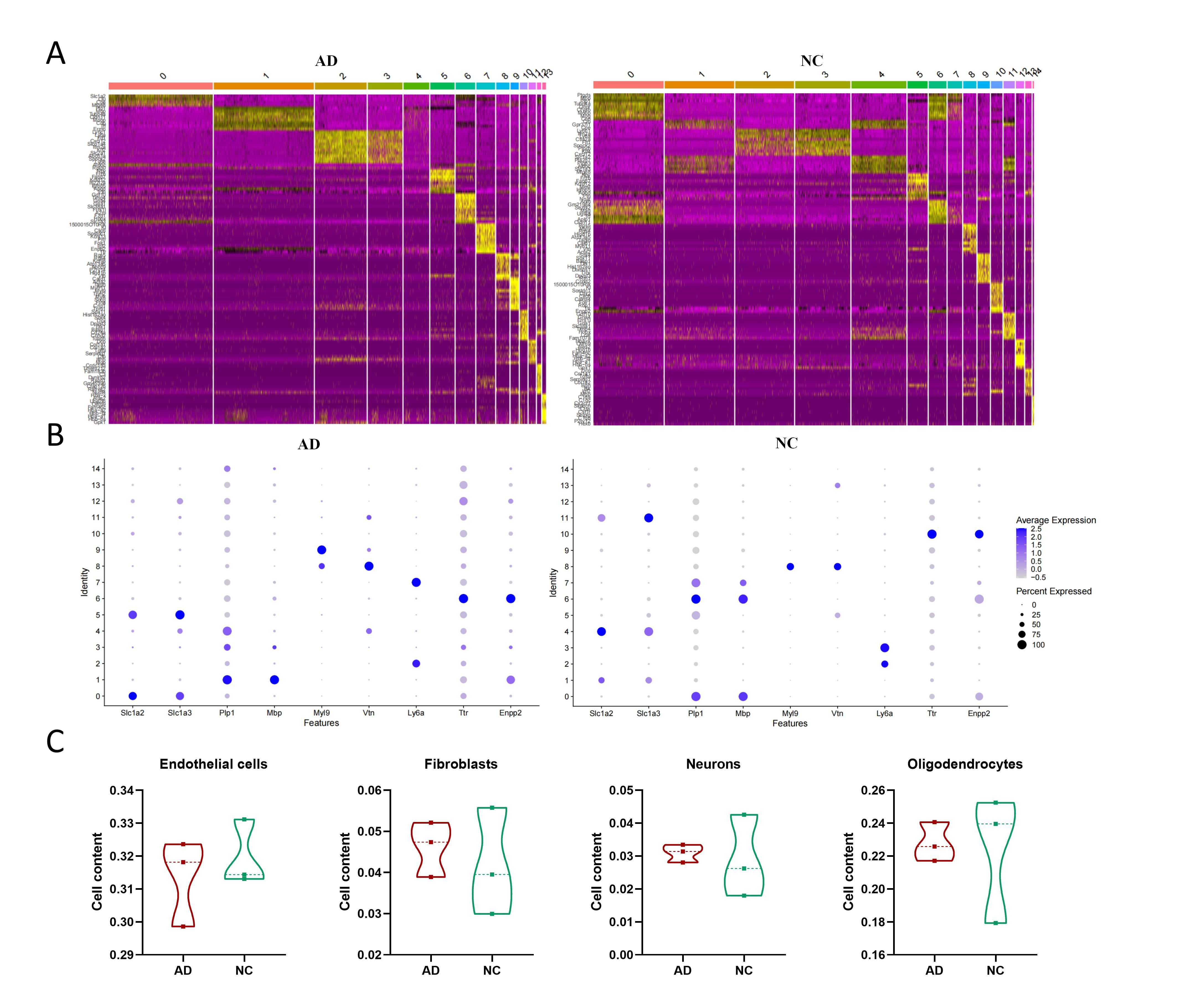

Supplement: Supplementary file 3 — Figure S3. Analysis of cell clustering results in scRNA‐seq data. (A) heatmap displaying the top 10 marker gene expressions in each cluster; (B) bubble plot showing the expression pattern of selected marker genes in each cluster; and (C) T‐test results indicating no statistical difference in the cell type between the AD group (N = 3) and NC group (N = 3). [file CNS-31-e70338-s003.jpg]

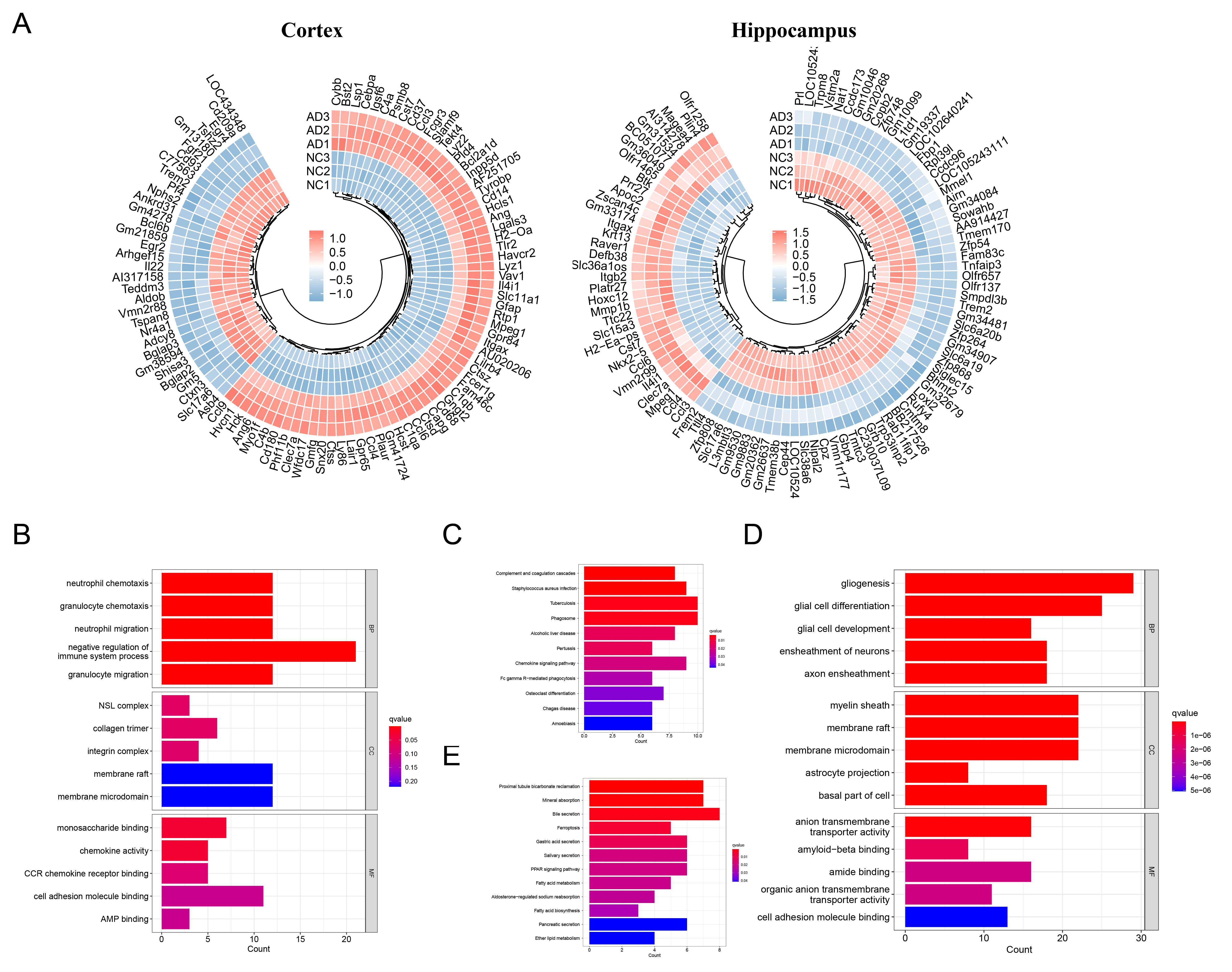

Supplement: Supplementary file 4 — Figure S4. Differential analysis and functional enrichment analysis of transcriptome sequencing results. (A) circular heatmap showing the differential expression profiles of the top 100 genes with the most differential expression in the transcriptome sequencing results of the cerebral cortex and hippocampal tissues in the AD group (N = 3) compared to the NC group (N = 3); (B) bar plot of GO functional enrichment analysis for the intersection of differentially expressed genes, with the top five pathways ranked in biological processes (BP), cellular components (CC), and molecular functions (MF); (C) bar plot of KEGG pathway enrichment analysis for the intersection of differentially expressed genes; (D) bar plot of GO functional enrichment analysis for marker genes of astrocytes; and (E) bar plot of KEGG pathway enrichment analysis for marker genes of astrocytes. [file CNS-31-e70338-s005.jpg]

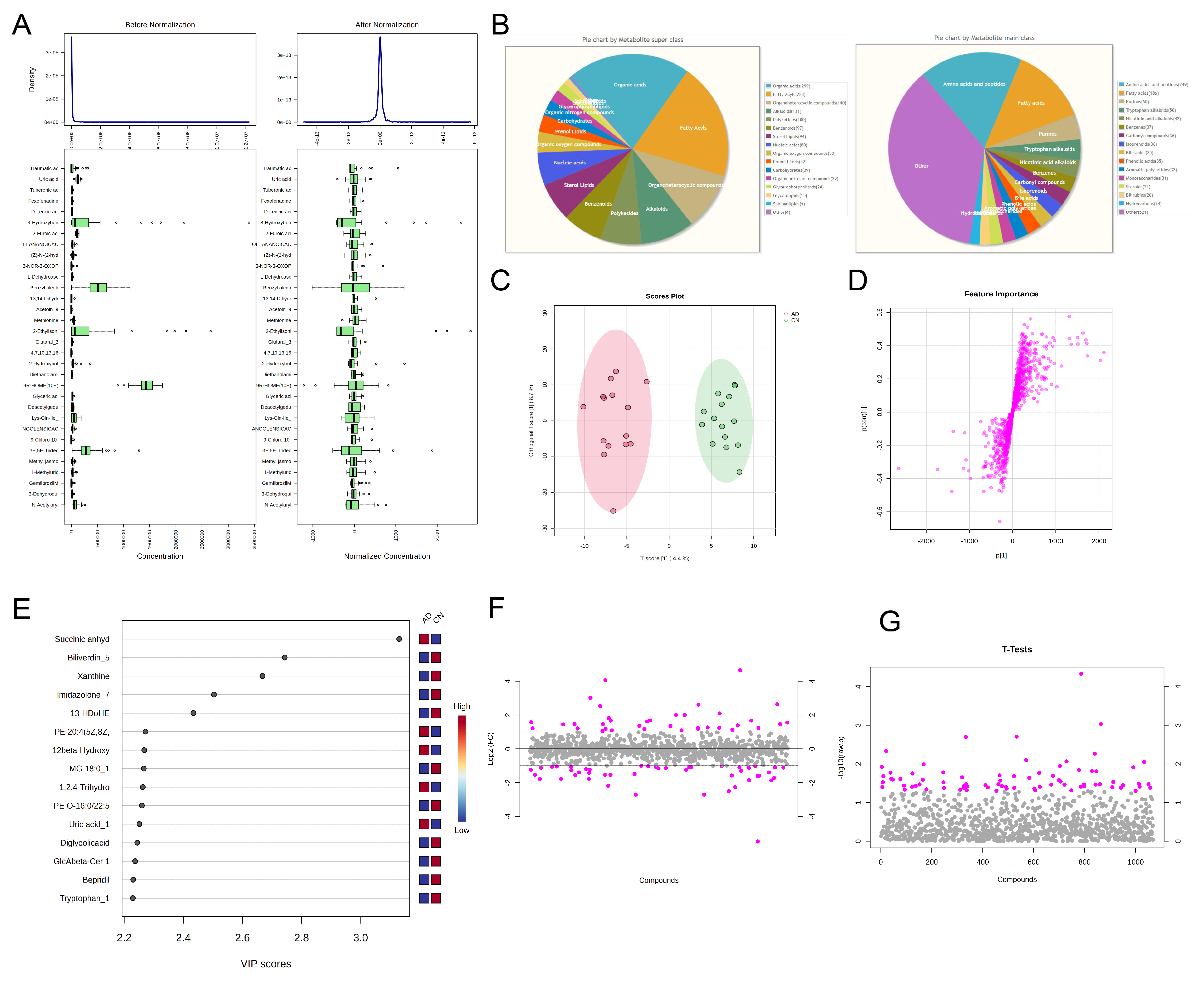

Supplement: Supplementary file 5 — Figure S5. Quality control and multivariate statistical analysis of metabolomic data. (A) comparison of raw metabolomics data before and after correction and normalization; (B) pie charts showing the composition of metabolites categorized based on “Super class” and “Main class”; (C) score plot of OPLS‐DA analysis, with the horizontal and vertical axes representing the scores of the primary component and orthogonal component, respectively, representing inter‐group and intra‐group differences; (D) S‐plot of OPLS‐DA analysis, with metabolites closer to the corners indicating higher importance in the model; (E) VIP values of top‐ranking metabolites based on OPLS‐DA analysis; (F) score plot of fold change analysis, with red dots representing |logFC| > 2 in comparisons between the two groups; and (G) T‐test analysis results, with red dots representing p < 0.05 in comparisons between the two groups. Each group contains N = 6 samples. [file CNS-31-e70338-s002.jpg]

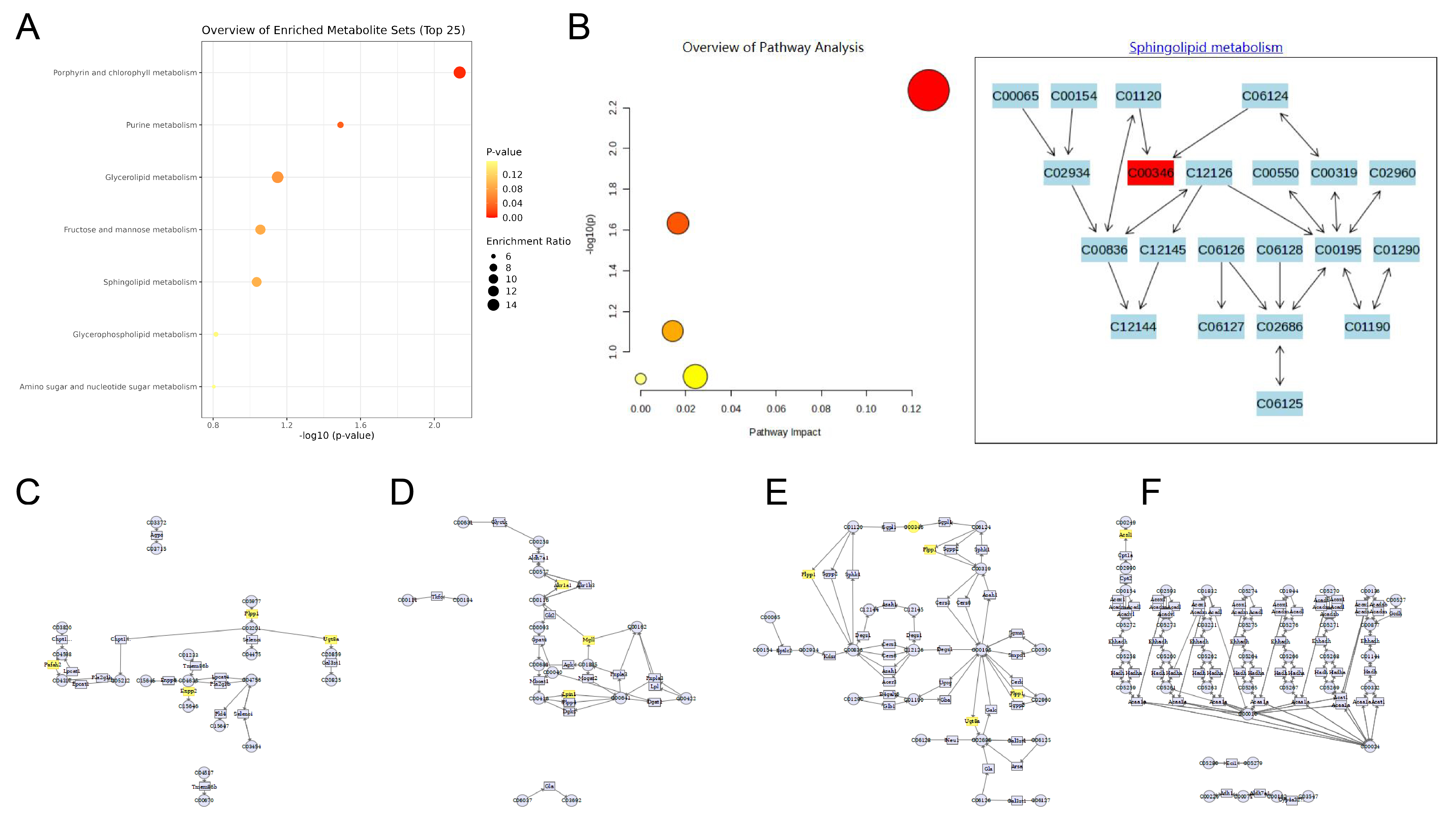

Supplement: Supplementary file 6 — Figure S6. Functional and pathway enrichment analysis of differential metabolites. (A) bubble plot of functional enrichment analysis results of differential metabolites in the MetaboAnalyst database; (B) pathway enrichment analysis results of differential metabolites in the MetaboAnalyst database, with the display of the “Sphingolipid metabolism” pathway and enriched metabolites on the right; and (C–F) the “Ether lipid metabolism,” “Glycerolipid metabolism,” “Sphingolipid metabolism,” and “Fatty acid degradation” pathways in the integrated metabolic pathway analysis results. [file CNS-31-e70338-s001.jpg]

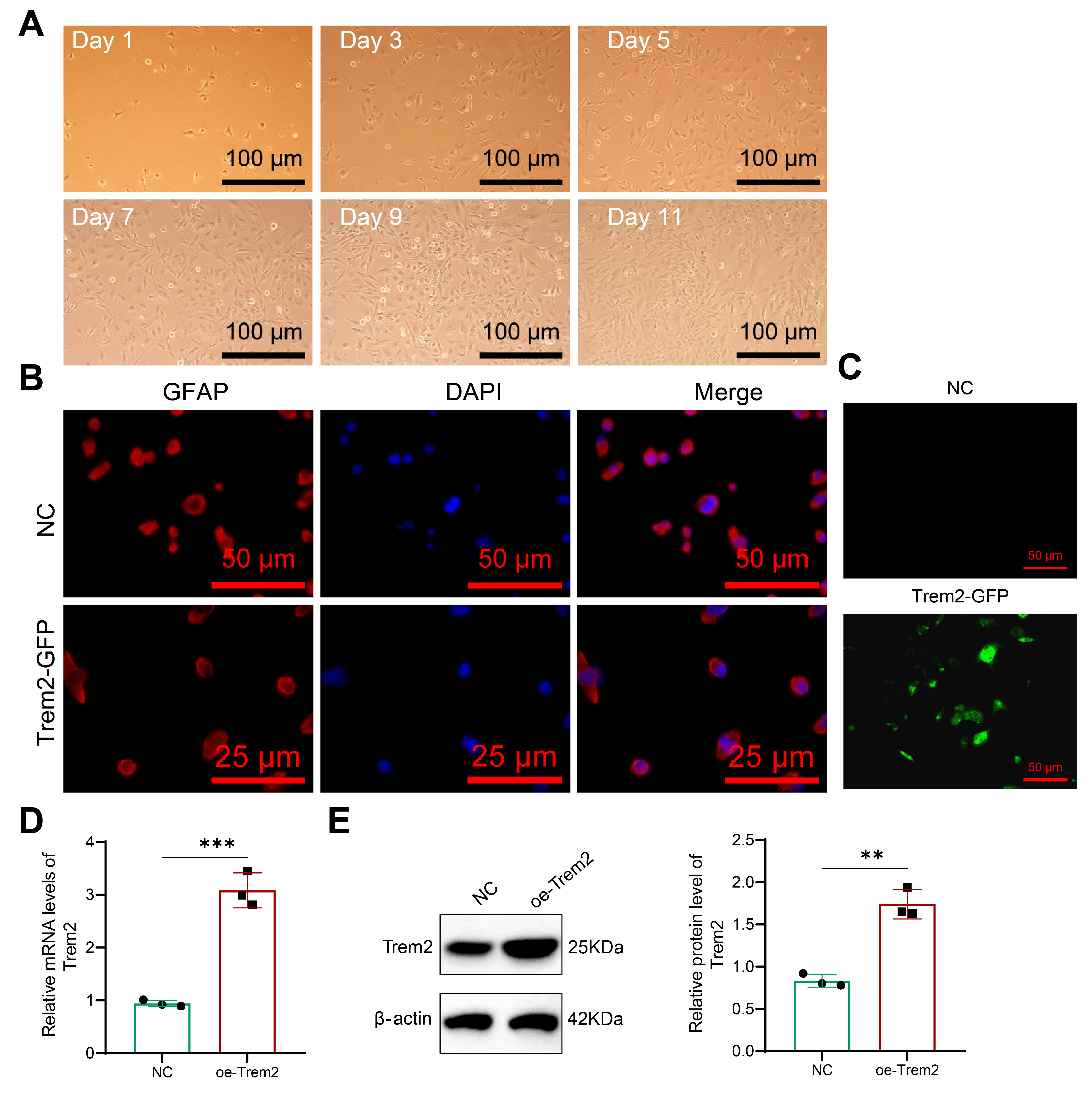

Supplement: Supplementary file 7 — Figure S7. Identification of astrocytes and transfection assessment. (A) Changes in growth conditions of primary astrocytes observed under an inverted microscope on days 1, 3, 5, 7, 9, and 11; (B) immunofluorescence staining of specific astrocytic marker GFAP; (C) observation of Trem2 overexpression plasmid transfection effect under a microscope; (D) expression level of Trem2 in transfected cells detected by RT‐qPCR; and (E) expression level of Trem2 protein in transfected cells detected by western Blot. **p < 0.01, ***p < 0.001 between the two groups, N = 3. NC (normal control): cells from non‐treated, normal astrocytes; Oe‐Trem2 (overexpressed Trem2): astrocytes that were transfected to overexpress Trem2 to study the effects of Trem2 overexpression on inflammation. [file CNS-31-e70338-s004.jpg]

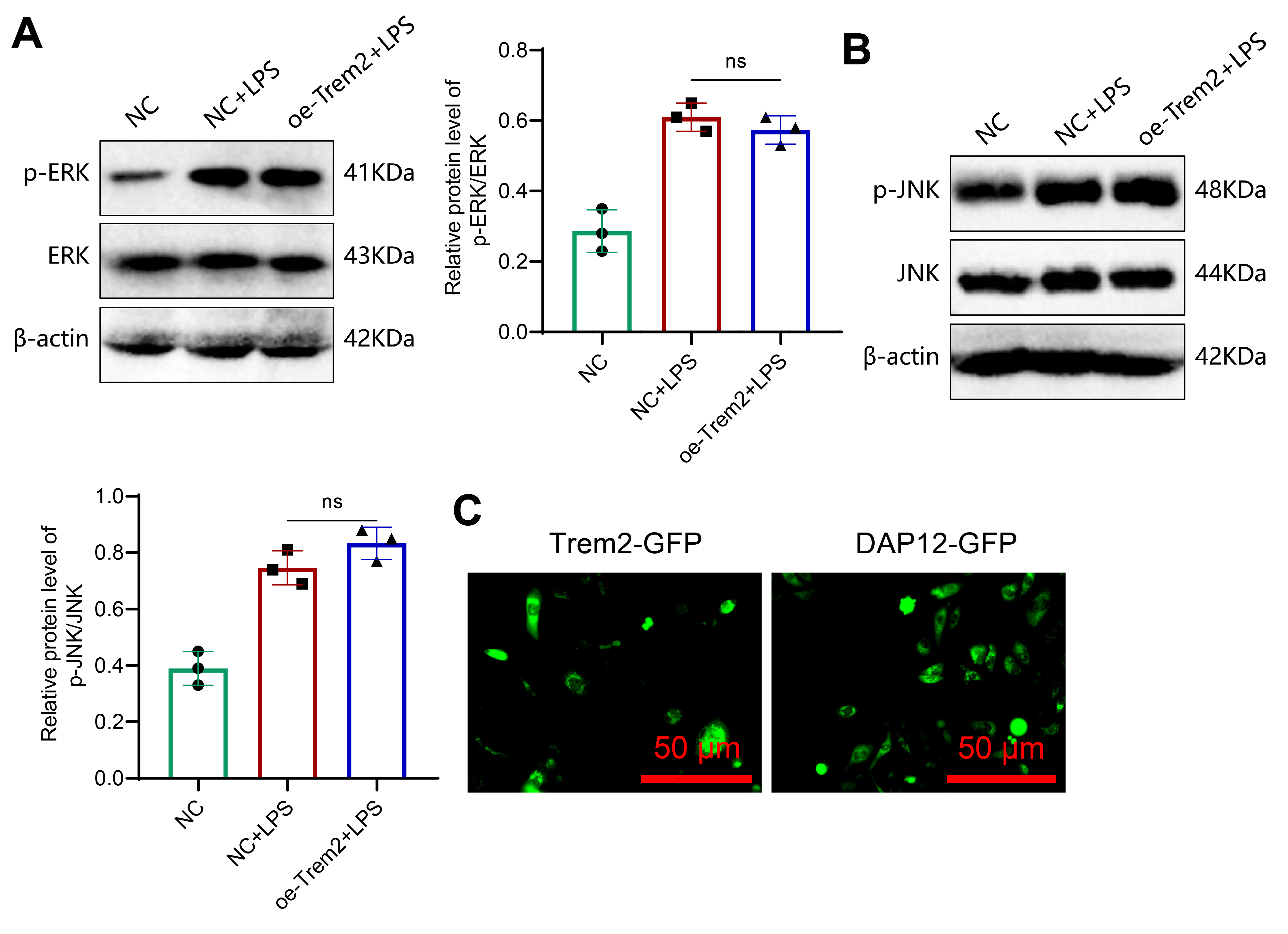

Supplement: Supplementary file 8 — Figure S8. Mechanistic study of Trem2’s impact on inflammatory factor production. (A) Expression and activation levels of key downstream signaling molecule ERK in TLR4‐activated cells detected by western Blot; (B) expression and activation levels of key downstream signaling molecule JNK in TLR4‐activated cells detected by western Blot; and (C) observation of Trem2 and DAP12 overexpression plasmid transfection effect under a microscope. NS represents no statistical difference between the two groups, N = 3. [file CNS-31-e70338-s010.jpg]

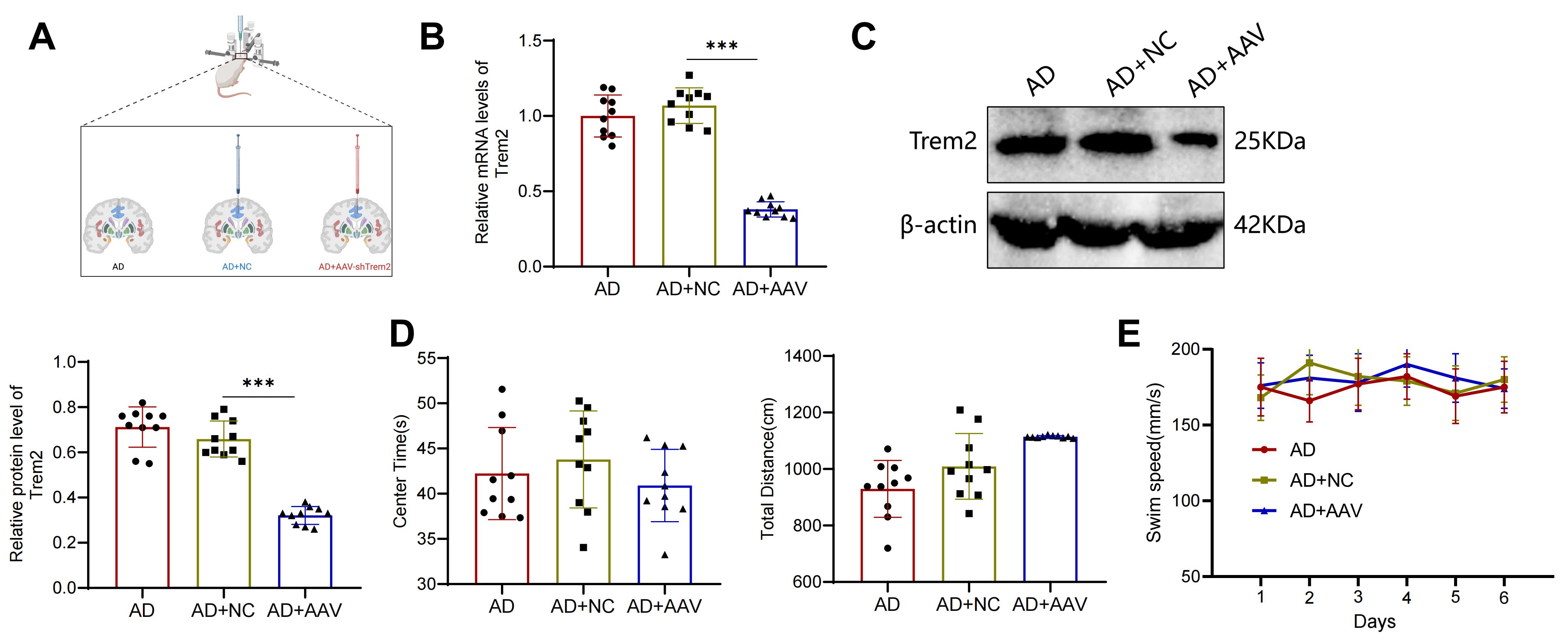

Supplement: Supplementary file 9 — Figure S9. Effects of Trem2 knockdown on mouse AD behavior. (A) Schematic representation of stereotaxic injection of Trem2 shRNA into the mouse brain to interfere with Trem2 expression in AD mice; (B) mRNA expression level of Trem2 in the mouse hippocampus detected by RT‐qPCR; (C) protein expression level of Trem2 in the mouse hippocampus detected by western Blot; (D) time spent in the center area and total distance traveled by mice in the open field test; and (E) swimming speed of mice during the 6‐day water maze test. *p < 0.05 between the two groups, N = 10. [file CNS-31-e70338-s009.jpg]
