# Supplementary material for: Elucidating the Role of Trem2 in Lipid Metabolism and Neuroinflammation
Source: CNS Neurosci Ther. 2025 Apr 9;31(4):e70338. doi: 10.1111/cns.70338 (PMC11982525; doi:10.1111/cns.70338)
Supplement: Supplementary file 10 — Table S1. Antibody information. Table S2. Primer sequence. Table S3. Clustering analysis and cell annotation results of AD group. Table S4. Clustering analysis and cell annotation results of NC group. Table S5. Proportion of different types of cells in AD and NC groups. [file CNS-31-e70338-s007.docx]

| Antibody/Kit | Product No. | Dilution ratio | Manufacturer |
| --- | --- | --- | --- |
| Rabbit monoclonal to TREM2 | ab305103 | 1:1000 | Abcam |
| Rabbit monoclonal to TNF-α | ab183218 | 1:1000 | Abcam |
| Rabbit monoclonal to IL-6 | ab290735 | 1:1000 | Abcam |
| Rabbit monoclonal to IL-1β | ab254360 | 1:1000 | Abcam |
| Rabbit monoclonal to NF-kB p65 (phospho S536) | ab76302 | 1:1000 | Abcam |
| Rabbit monoclonal to NF-kB p65 | ab32536 | 1:1000 | Abcam |
| Rabbit monoclonal to ERK1 (phospho T202) | ab201015 | 1:1000 | Abcam |
| Rabbit monoclonal to ERK1 | ab32537 | 1:1000 | Abcam |
| Rabbit monoclonal to JNK1 (phospho T183 + Y185) | ab307802 | 1:1000 | Abcam |
| Rabbit monoclonal to JNK1 | ab199380 | 1:2500 | Abcam |
| Mouse monoclonal to beta Actin | ab8226 | 1:1000 | Abcam |
| Goat Anti-Rabbit IgG | ab150077 | 1:5000 | Abcam |
| Goat Anti-Mouse IgG | ab150113 | 1:5000 | Abcam |
| Mouse TNF-α ELISA Kit | ab208348 | \ | Abcam |
| Mouse IL-6 ELISA Kit | ab222503 | \ | Abcam |
| Mouse IL-1β ELISA Kit | ab197742 | \ | Abcam |
| Anti-NF-kB p65 IF | ab32536 | 1:100 | Abcam |
| Anti-TREM2 IF | ab305103 | 1:1000 | Abcam |
| Anti-S100 beta - Astrocyte Marker IF | ab52642 | 1:100 | Abcam |
| Anti-GFAP IF | ab7260 | 1:5000 | Abcam |
| DAPI IF | ab285390 | 1:500 | Abcam |

**Table S1. Antibody information**

**Table S2. Primer sequence**

| Gene | Primer sequence (5’-3’) |
| --- | --- |
| Trem2(mouse) | Forward: TCATCGAGTTTCGAGGGTGC |
|  | Reverse: TGGTAGGCTAGAGGTGACCC |
| TNF-α(mouse) | Forword: GATCGGTCCCCAAAGGGATG |
|  | Reverse: CCACTTGGTGGTTTGTGAGTG |
| IL-6(mouse) | Forword: TGGTCTTCTGGAGTACCATAGC |
|  | Reverse: TGTGACTCCAGCTTATCTCTTGG |
| IL-1β(mouse) | Forword: TGCCACCTTTTGACAGTGATG |
|  | Reverse: TGATGTGCTGCTGCGAGATT |
| β-actin(mouse) | Forword: CACTGTCGAGTCGCGTCC |
|  | Reverse: CGCAGCGATATCGTCATCCA |

**Table S3. Clustering analysis and cell annotation results of AD group**

| Cell type | Clusters | Marker gene |
| --- | --- | --- |
| Astrocytes | Cluster 0, 6 | Slc1a2, Slc1a3, App |
| Endothelial cells | Cluster 2, 3, 8 | Ly6a, Ly6c1, Flt1 |
| Epithelial cells | Cluster 7, 12 | Kl, Clic6 |
| Fibroblasts | Cluster 9, 11 | Tagln, Enpp2 |
| Neurons | Cluster 10 | Sox11 |
| Oligodendrocytes | Cluster 1, 4, 5, 13 | Plp1, Mbp, Qdpr |

**Table S4. Clustering analysis and cell annotation results of NC group**

| Cell type | Clusters | Marker gene |
| --- | --- | --- |
| Astrocytes | Cluster 1, 4, 11 | Slc1a2, Slc1a3, Plpp3, Apoe |
| Endothelial cells | Cluster 2, 3, 8 | Ly6a, Flt1 |
| Epithelial cells | Cluster 10 | Kl, Sostdc1, Otx2 |
| Erythrocytes | Cluster 12 | Olfr69, Alas2 |
| Fibroblasts | Cluster 13 | Dcn, Col1a1 |
| Microglia | Cluster 14 | C1qa, Cx3cr1 |
| Neurons | Cluster 9 | Sox4, Sox11 |
| Oligodendrocytes | Cluster 0, 5, 6, 7 | Plp1, Mbp, Tubb4a |

**Table S5. Proportion of different types of cells in AD and NC groups**

| Cell type | AD | NC | P value* |
| --- | --- | --- | --- |
| Astrocytes | 34.41% | 30.89% | < 0.05 |
| Endothelial cells | 31.92% | 31.15% | NS |
| Epithelial cells | 3.75% | 7.82% | < 0.05 |
| Fibroblasts | 5.01% | 4.13% | NS |
| Neurons | 2.89% | 2.70% | NS |
| Oligodendrocytes | 22.32% | 23.24% | NS |

*NS represents no statistically significant difference between the two groups.
